# The Clinical Impact of the EPH/Ephrin System in Cancer: Unwinding the Thread

**DOI:** 10.3390/ijms22168412

**Published:** 2021-08-05

**Authors:** Alexandros Pergaris, Eugene Danas, Dimitrios Goutas, Alexandros G. Sykaras, Angelos Soranidis, Stamatios Theocharis

**Affiliations:** First Department of Pathology, Medical School, National and Kapodistrian University of Athens, 11527 Athens, Greece; alexperg@yahoo.com (A.P.); eugenedanas@gmail.com (E.D.); dimgoutas@med.uoa.gr (D.G.); alexander.sykaras@gmail.com (A.G.S.); angelossoranidis@hotmail.com (A.S.)

**Keywords:** EPHs, ephrins, biomarkers, cancer, diagnosis, prognosis, treatment

## Abstract

Erythropoietin-producing human hepatocellular receptors (EPHs) compose the largest known subfamily of receptor tyrosine kinases (RTKs). They bind and interact with the EPH family receptor interacting proteins (ephrins). EPHs/ephrins are implicated in a variety of physiological processes, as well as in cancer pathogenesis. With neoplastic disease remaining a leading cause of death world-wide, the development of novel biomarkers aiding in the field of diagnosis, prognosis, and disease monitoring is of utmost importance. A multitude of studies have proven the association between the expression of members of the EPH/ephrin system and various clinicopathological parameters, including disease stage, tumor histologic grade, and patients’ overall survival. Besides their utilization in timely disease detection and assessment of outcome, EPHs/ephrins could also represent possible novel therapeutic targets. The aim of the current review of the literature was to present the existing data regarding the association between EPH/ephrin system expression and the clinical characteristics of malignant tumors.

## 1. Introduction

Neoplastic disease remains a leading cause of death, with approximately 19.3 million new cancer cases diagnosed in 2020 worldwide, accompanied by an estimated 10 million cancer-related deaths [1]. Furthermore, according to the National Cancer Institute, an estimated 39.5% of men and women will be diagnosed with cancer at some point during their lifetime [2]. Such statistics indicate the heavy impact of neoplasia on patients’ welfare, healthcare systems, and socio-economic stability. Despite ongoing advances in therapeutic interventions, further research is called for to combat the immense challenge imposed by neoplasia. Modern studies continue to shed light on the molecular mechanisms implicated in key steps of cancer pathogenesis, such as tumor formation and growth, invasion, lymph- and angiogenesis, as well as distant dissemination. The development of novel, specialized treatment strategies renders the understanding of these molecular pathways and their clinical impact of paramount importance. Furthermore, the well-established positive effect of early therapeutic intervention on the outcome of cancer patients underlines the need for the discovery of diagnostic, prognostic and predictive biomarkers contributing to timely detection of disease progression and re-currence, as well as assessment of therapeutic outcome. Scientists have shifted their focus on molecules participating in physiologic processes that, when deregulated, can enhance carcinogenesis. Among them, EPHs, with their well-established roles in proliferation, angiogenesis, and cell motility, are speculated to represent key factors in cancer pathogenesis. Therefore, a great number of studies attempted to assess their utilization as diagnostic and prognostic biomarkers, as well as therapeutic targets.

**Figure 1:**
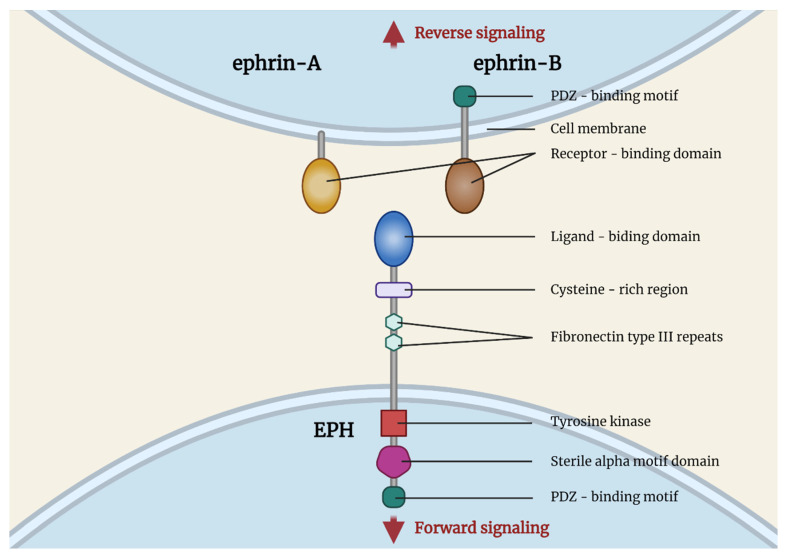
Structure of EPHs/ephrins. Created with BioRender.com (accessed on 2 August 2021).

EPHs compose the largest known subfamily of receptor tyrosine kinases. They bind and interact with the EPH family receptor interacting proteins (ephrins). EPHs are membrane-bound proteins, consisting of an extracellular ephrin-binding domain, a transmembrane region, and a cytoplasmic component that, upon activation, following interaction with a ligand, initiates its RTK activity, activating downstream signaling molecular pathways. Ligand binding activation requires cell-to-cell interaction. Subsequently, as a response, signaling cascades are triggered in the cytoplasm of both the EPH-expressing cell and the ephrin-bearing one, a process termed as backward signaling (Figure 1). Nine EPHA receptors (EPHA1 to EPHA8 and EPHA10), that bind 5 ephrin-A ligands (ephrin-A1 to ephrin-A5), along with 5 EPHB receptors (EPHB1 to EPHB4 and EPHB6), that interact with 3 ephrin-B ligands (ephrin-B1 to ephrin-B3), are expressed in humans. EPHs of a specific subgroup (A or B) show a higher affinity for binding with ephrins belonging to the same subgroup, although inter-subgroup interaction has been frequently observed.

EPHs/ephrins participate in wide variety of processes in human physiology. They play a pivotal role in cell migration, axon guidance, and synapse formation during embryonic development. They are also implicated in mechanisms like cell adhesion, motility, and cell-matrix interactions, as well as lymphangiogenesis and angiogenesis in response to hypoxia [3].

All the aforementioned processes are vital for tumorigenesis, as cell motility is required for cancer cells’ invasion, vessel infiltration, and spreading to distant sites. Moreover, tumors cannot grow beyond a few millimeters without the sprouting of new vessels to provide them with oxygen and nutrients [4]. As accumulating data clearly underlined the clinical implications of EPH/ephrin tumor profiles, many in vivo and in vitro studies focused on revealing the molecular mechanisms through which members of the EPH/ephrin family exert their tumor promoting and tumor suppressive properties. In glioblastoma cell lines, EPHB4, along with its ligand, ephrin-B2, were observed to enhance angiogenesis through interacting with VGFR2 and Notch signaling pathways [5]. EPHA3, on the contrary, seems to enhance apoptosis of lung carcinoma cells via phosphorylation of members of the PI3K/BMX/STAT3 signaling pathway [5]. In breast cancer cells, phosphorylation of EPHA3 via Src kinase, due to trastuzumab treatment, induces the activation of PI3K/Akt and MAPK pathways, leading to trastuzumab resistance [5].

**Figure 2:**
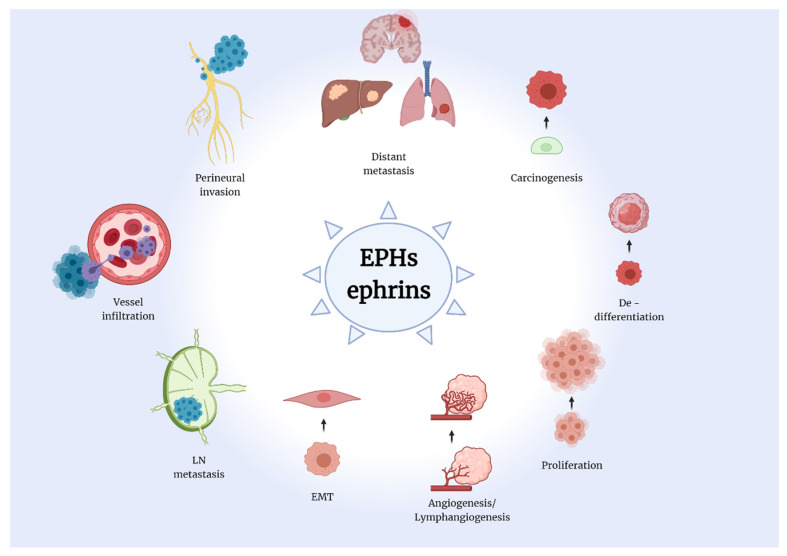
The Eph/ephrin system is implied in a multitude of tumorigenic processes. EPH: erythropoietin-producing human hepatocellular receptors, ephrins: EPH family receptor interacting proteins, EMT: epithelial-mesenchymal transition, LN: lymph nodes. Created with BioRender.com (accessed on 2 August 2021).

Therefore, the implication of the EPH/ephrin system in cell motility, cell-to-cell and cell-matrix interaction, as well as lymph- and angiogenesis renders them a possible key factor in carcinogenesis and an excellent biomarker candidate of tumor promoting and suppressive properties (Figure 2). Indeed, a plethora of studies have attempted to reveal the link between EPH/ephrins and tumorigenesis, as well as tumor characteristics and behavior. Specifically, in solid tumors, researchers have proven the close relationship between the various members of the EPH/ephrin system expression and disease onset and progression. Moreover, therapeutic agents that suppress those mechanisms could represent excellent possible targets for the development of novel, specialized anti-cancer intervention strategies.

In the current review, data regarding the association between EPH/ephrin expression and the clinical characteristics of neoplasia were presented.

## 2. Methodology

We searched, via the pubmed engine, information regarding the role of the members of the EPH/ephrin family in carcinogenesis of solid tumors, focusing on the clinical impact of their expression. The majority of researchers investigated the protein expression of EPHs/ephrins through immunohistochemistry (IHC), IHC in tissue microarrays (TMA IHC), and Western Blot, as well as their mRNA expression, utilizing methods such as the Polymerase Chain Reaction (PCR), reverse-transcription PCR (RT-PCR), and quantitative PCR (q PCR), in a series of tumor specimens from patients with malignant disease. The presence or absence of expression was assessed, as well as the location (nuclear, cytoplasmic or membranous), the staining intensity, and the percentage of positive cells to overall tumor cell population. In addition, observations were made regarding different patterns of expression, such as diffuse staining, positive clusters of cells, and increased staining intensity on the tumor invasive front in deeper normal tissue layers. Moreover, most studies investigated the differences in EPHs/ephrins expression by including normal and non-neoplastic tissue samples, either from tissues adjacent to the malignant ones or from healthy individuals. The researchers then proceeded to associate their observations on EPHs/ephrins gene and protein expression with a wide range of clinicopathological parameters, including disease stage, histological grade, presence of lymph node (LN) or distant metastasis, overall survival (OS), and disease-free survival (DFS). Furthermore, the patterns of EPH/ephrin system members expression were correlated to a variety of histological characteristics, such as invasion of vessels, perineural infiltration, Microvessel Density (MVD), level of tumor neo-angiogenesis, and lymphangiogenesis and tumor cell proliferative capacity.

## 3. Head and Neck Tumors

### 3.1. Brain

The impact of the EPH/ephrin system expression in brain tumors is among the most extensively studied, with results suggesting that the aforementioned biomolecules play an overwhelmingly tumor-promoting role on brain cancer pathogenesis [6,7,8,9,10,11,12].

#### 3.1.1. EPHAs

EPHA2 expression was assessed in normal brain samples and glioma tissues through either IHC or RT-PCR in three studies [6,7,8]. EPHA2 expression was then correlated with tumor histological features, various clinicopathological parameters, and patients’ outcomes. EPHA2 overexpression was positively linked to tumor size [8], higher histological grade of tumors [6,8], peritumoral edema levels [8], and poor patients prognosis [7,8]. EPHA4 expression, on the other hand, exhibited no significant difference between glioblastoma multiforme (GBM) and non-tumoral white matter tissue [9]. EPHA7 immunohistochemical expression was notably absent in the 10 normal brain tissues included in the study and strong in 14 out of 32 cases of primary or recurrent GBM tissues investigated. EPHA7 expression was positively associated with tumor MVD, increased age, and decreased OS [10].

#### 3.1.2. EPHBs

In a large study that included 171 tumor samples (29 astrocytomas, 82 GBM, 49 oligodendrogliomas, 11 oligoastrocytomas) and 24 non-tumorous brain specimens from epileptogenic patients, EPHB1, B2, B3, B4, and B6 expression was assessed by quantitative real-time RT-PCR [11]. EPHB1 seemed to have a beneficial influence, since its expression correlated with longer patient OS and was upregulated in oligodendrogliomas, which notably have a more favorable outcome compared to other infiltrating glioma tumors. EPHB2 and EPHB3 expression was reported as higher in neoplastic tissues compared to normal ones, and EPHB4 expression was correlated with shorter patient OS. EPHB4’s negative impact was also underlined in another series of 96 primary glioma patients [12], as its expression was positively associated with higher grade and lower Karnofsky Performance Score. Interestingly, EPHB6, along with EPHB1, seems to suppress tumorigenesis. In the aforementioned study by Teng et al [11], EPHB6 expression was reported as lower in neoplastic tissues compared with non-tumorous ones.

#### 3.1.3. Ephrins

Ephrin-A1 expression was associated with lower grade gliomas, while patients with tumors positive for EPHA2 and negative for ephrin-A1 exhibited shorter OS and PFS. On the other hand, Tu et al correlated the elevated ephrin-B2 expression to higher-grade tumors and low Karnofsky Performance Score and designated it as an independent negative prognostic factor for progression-free survival of glioma patients [12]. Likewise, ephrin-B3 seems to negatively impact Central Nervous System neoplasia, with its expression in gliomas reported 2.5-fold higher in tumor samples compared with controls and linked to higher grade tumors [9]. Information concerning the clinical impact of EPH/ephrin expression in brain tumors is summarized in Table 1.

### 3.2. Salivary Glands

In the single study of EPH/ephrin expression in salivary gland tumors, EPHA2 and ephrin-A1 expression were assessed by IHC, Western blot, and real-time RT PCR in 49 primary Adenoid Cystic Carcinomas (ACCs) and 10 normal salivary gland tissues [13]. EPHA2 and ephrin-A1 expression was reported as higher in ACC tissues compared with non-neoplastic ones, and positively correlated with increased MVD. EPHA2 and ephrin-A1 overexpression, along with elevated MVD, was associated with the tumor TNM stage and the presence of perineural and perivascular invasion. Of note, solid type ACCs, which are accompanied by worse prognoses, exhibited higher EPHA2 and ephrin-A1 expression and increased MVD compared with cribriform and tubular subtypes [13].

**Figure 3:**
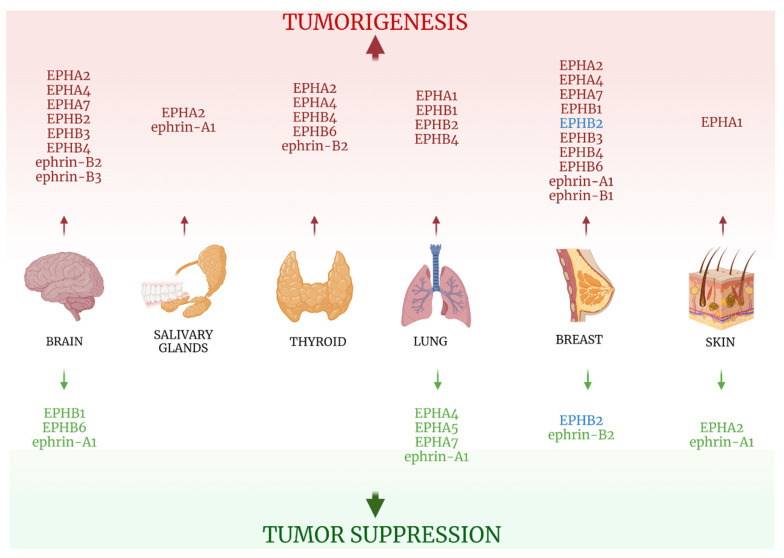
EPH/ephrin family role in solid tumors of the brain, salivary glands, thyroid, lung, breast, and skin. Red-colored EPHs/ephrins enhance tumorigenesis, while green-colored ones suppress it. EPHB2 in breast cancer can play both roles, depending on its location: cytoplasmic EPHB2 expression promotes carcinogenesis, while membranous EPHB2 expression favors tumor-suppression. Created with BioRender.com (accessed on 2 August 2021).

Fukai et al reported a case of a 29-year-old male ACC patient that presented with perineural spread of the tumor along the mandibular nerve [14]. Tumor cells showed characteristics of Epithelial-Mesenchymal Transition (EMT) and a high expression of EPHA2 (but not ephrin-A1), using IHC, suggesting a possible link between the receptor and aggressive tumor phenotypes. Table 1 and Figure 3 summarize the data concerning EPH/ephrin expression and salivary gland tumors clinicopathological characteristics.

### 3.3. Thyroid

#### 3.3.1. EPHAs

EPHA2 expression was reported as higher in thyroid carcinoma compared with non-cancerous thyroid tissue in two studies [15,16], although no further association with clinicopathological parameters was noted, while EPHA4 showed no difference in expression levels at all [15].

#### 3.3.2. EPHBs

In a study that enrolled 46 papillary and 10 follicular thyroid carcinoma tissues, as well as 71 benign thyroid samples, EPHB4 expression was found to be higher in malignant specimens compared with their benign counterparts, while its overexpression was associated with larger tumor sizes [17]. In consistency with those findings, Sharma et al also correlated EPHB4 increased expression with malignancy, as well as with the presence of LN metastasis [18]. It is noteworthy that increased expression of an EPH may hinder tumorigenesis of a certain type of tumor but promote carcinogenesis of another one. The study conducted by Giaginis et al [17] underlined the association between elevated EPHB6 expression and malignancy, tumor size, presence of LN metastasis, capsular, vascular, and lymphatic invasion, as well as increased recurrence rate. Those results are in contrast with the beneficial role of EPHB6 in glioma pathogenesis [11].

#### 3.3.3. Ephrins

Ephirn-B2 protein overexpression was correlated with malignant status and LN metastasis [18], while its elevated *mRNA* expression was linked to higher TNM stage cases [19].

All information on EPH/ephrin expression on thyroid neoplastic tissues is summarized in Table 1 and Figure 3.

**Table 1:** EPHs/ephrins (bold) studied in solid tumors of head and neck and correlations with clinicopathological parameters.

EPHs/Ephrins	Malignant Tissues	Benign Control Tissues	Methods	Results	Refs
**BRAIN**					
**EPHA2**	78 primary glioma samples		IHC	**EPHA2** overexpression associated with: ○ higher grade histology ○ poor OS Patients with tumors positive for **EPHA2** and negative for ephrin-A1 had the shorter OS and progression-free survival	[4]
	21 glioblastoma samples	5 non-tumorous human brain samples	RT-PCR real-time RT-PCR	***EPHA2 mRNA*** overexpression correlated inversely with patients’ OS	[5]
	43 glioma samples		IHC MRI	**EPHA2** expression higher in high-grade tumors **EPHA2** expression correlated with: ○ peritumoral edema index ○ tumor enhancement percentage ○ maximum tumor diameter	[6]
**EPHA4**	31 GBM samples	28 non-tumoral white matter samples	q RT-PCR	No significant change found in **EPHA4** expression in tumor tissues compared to normal ones	[7]
**EPHA7**	32 stage IV GBM samples (26 primary, 6 recurrent)	10 normal brain samples	IHC	**EPHA7** increased expression correlated with: ○ decreased OS ○ increased age ○ increased tumor MVD	[8]
**EPHB1**	171 tumor samples (29 astrocytomas, 82 GBM, 49 oligodendrogliomas, 11 oligoastrocytomas)	24 non-neoplastic brain samples from epileptogenic patients	q real-time RT-PCR	**EPHB1** levels did not vary between tumor and normal brain specimens (except for increased expression in oligodendrogliomas) High **EPHB1** expression levels linked to longer OS	[9]
**EPHB2**	171 tumor samples (29 astrocytomas, 82 GBM, 49 oligodendrogliomas, 11 oligoastrocytomas)	24 non-neoplastic brain samples from epileptogenic patients	q real-time RT-PCR	**EPHB2** expression higher in GBM tissues than normal brain specimens	[9]
**EPHB3**	171 tumor samples (29 astrocytomas, 82 GBM, 49 oligodendrogliomas, 11 oligoastrocytomas)	24 non-neoplastic brain samples from epileptogenic patients	q real-time RT-PCR	**EPHB3** expression higher in GBM tissues than normal brain specimens	[9]
**EPHB4**	171 tumor samples (29 astrocytomas, 82 GBM, 49 oligodendrogliomas, 11 oligoastrocytomas)	24 non-neoplastic brain samples from epileptogenic patients	q real-time RT-PCR	**EPHB4** expression higher in GBM tissues than normal brain specimens High **EPHB4** expression linked to shorter OS	[9]
	96 primary glioma samples		IHC	Expression of **EPHB4** correlated with expression of **ephrin-B2** High expression of **EPHB4** correlated with: ○ higher grade ○ lower Karnofsky Performance Score High **EPHB4** expression was an independent negative prognostic factor for progression-free survival in glioblastoma No association between **EPHB4** expression and age/ gender/ tumor location	[10]
**EPHB6**	171 tumor samples (29 astrocytomas, 82 GBM, 49 oligodendrogliomas, 11 oligoastrocytomas)	24 non-neoplastic brain samples from epileptogenic patients	q real-time RT-PCR	**EPHB6** weakly expressed in diffuse astrocytomas, anaplastic astrocytomas, and GBM compared with non-neoplastic brain tissues	[9]
**ephrin-A1**	78 primary gliomas samples		IHC	**ephrin-A1** overexpression associated with lower-grade histology Patients with tumors positive for **EPHA2** and negative for **ephrin-A1** had the shorter OS and progression-free survival	[4]
**ephrin-B2**	96 primary glioma samples		IHC	Expression of **ephrin-B2** correlated with expression of **EPHB4** High expression of **ephrin-B2** correlated with: ○ higher grade ○ lower Karnofsky Performance Score High **ephrin-B2** expression was an independent negative prognostic factor for progression-free survival in glioblastoma No association between **ephrin-B2** expression and age/gender/tumor location	[10]
**ephrin-B3**	31 GBM samples	28 non-tumoral white matter samples	q RT-PCR	**ephrin-B3** highly expressed in tumor samples (2.5-fold higher mean expression) compared with controls	[7]
	7 grade IV glioblastoma samples, 3 glioma samples (1 oligodendroglioma grade II, 1 astrocytoma grade II and 1 astrocytoma grade III)		IHC	**ephrin-B3** expression reported: ○ moderate to high in more than 70% of glioblastomas ○ rather low in all low-grade gliomas ○ notably focally high in perivascular areas	
**SALIVARY GLANDS**					
**EPHA2**	49 ACC samples	10 normal salivary gland samples	IHC Western blot real-time RT-PCR	**EPHA2** expression higher in ACC tissues **EPHA2** expression correlated with MVD **EPHA2** expression and MVD correlated with: ○ TNM stage ○ perineural invasion ○ perivascular invasion **EPHA2** expression and MVD greater in solid type ACC than in cribriform and tubular types	[11]
	1 case of ACC		IHC RT-PCR	Aberrant expression of EPHA2 reported. (EPHA2 suspected to play a part in EMT, as the tumor had mesenchymal IHC features)	[12]
**ephrin-A1**	49 primary ACC samples	10 normal salivary gland samples	IHC Western blot real-time RT-PCR	**ephrin-A1** expression higher in ACC tissues **ephrin-A1** expression correlated with MVD **ephrin-A1** expression and MVD correlated with: ○ TNM stage ○ perineural invasion ○ perivascular invasion **ephrin-A1** expression and MVD greater in solid type ACC than in cribriform and tubular types	[11]
	1 case of ACC		IHC RT-PCR	No expression of **ephrin-A1** reported	[12]
**THYROID**					
**EPHA2**	59 thyroid carcinoma samples (47 papillary, 5 follicular, 5 medullary, 2 anaplastic)	72 benign tissue samples (61 hyperplastic nodules, 11 Hashimoto thyroiditis samples)	IHC	**EPHA2** overexpressed in malignant tissues compared with benign ones **EPHA2** expression not associated with TNM stage or capsular/vascular/lymphatic invasion	[13]
	74 thyroid carcinoma samples (68 papillary, 6 follicular)	14 follicular adenoma samples	IHC	**EPHA2** significantly higher in cancer tissues than normal ones	[14]
**EPHA4**	59 thyroid carcinoma samples (47 papillary, 5 follicular, 5 medullary, 2 anaplastic)	72 benign tissue samples (61 hyperplastic nodules, 11 hashimoto thyroiditis samples)	IHC	**EPHA4** expression not differentiated between malignant and benign cases Papillary carcinoma showed increased **EPHA4** expression compared with hyperplastic nodules **EPHA4** expression not associated with TNM stage or capsular/vascular/lymphatic invasion	[13]
**EPHB4**	56 thyroid carcinoma samples (46 papillary, 10 follicular)	71 benign thyroid tissues (56 hyperplastic nodules, 12 hashimoto thyroiditis tissues)	IHC	**EPHB4** overexpressed in malignant compared with benign tissues **EPHB4** expression provided a distinct discrimination between papillary carcinoma and hyperplastic nodules In malignant tumors, **EPHB4** overexpression associated with tumor size	[15]
	21 papillary carcinoma samples	surrounding normal thyroid samples	cDNA microarray Western blot IHC	**EPHB4** overexpression reported in tumor vs normal tissues **EPHB4** overexpression associated with presence of LN metastasis	[16]
**EPHB6**	56 thyroid carcinoma samples (46 papillary, 10 follicular)	71 benign thyroid samples (56 hyperplastic nodules, 12 hashimoto thyroiditis samples)	IHC	**EPHB6** overexpressed in malignant compared to benign tissues **EPHB6** expression provided a distinct discrimination between papillary carcinoma and hyperplastic nodules **EPHB6** overexpression associated with tumor size, positive LNs, capsular/vascular/lymphatic invasion and recurrence rate	[15]
**ephrin-B2**	21 papillary carcinoma samples	surrounding normal thyroid samples	cDNA microarray Western blot IHC	**ephrin-B2** overexpression reported in tumor vs normal tissues **ephrin-B2** overexpression associated with presence of LN metastasis	[16]
	66 malignant thyroid samples (23 follicular carcinomas, 18 follicular variant of papillary carcinomas, 25 papillary carcinomas)	4 normal thyroid samples, 26 hyperplastic nodules, 27 follicular adenomas	q real-time PCR	***ephrin-B2*** mRNA expression elevated in higher TNM stage neoplasms	[17]

IHC: immunohistochemistry, RT-PCR: reverse transcription-polymerase chain reaction, q RT-PCR: quantitative reverse transcription-polymerase chain reaction, OS: overall survival, GBM: glioblastoma multiforme, MVD: microvessel density, ACC: adenoid cystic carcinoma, EMT: epithelial-mesenchymal transition, cDNA: complementary DNA, LN: lymph nodes.

## 4. Thoracic and Skin Tumors

### 4.1. Lung

The role of EPH/ephrin system in lung carcinogenesis has been extensively studied in a number of works that incorporated a large numbers of specimens.

#### 4.1.1. EPHAs

EPHA1 overexpression was associated with high tumor proliferative capacity, assessed by the Ki67 index, probably revealing a link between its upregulation with more aggressive tumor phenotypes [20]. Intriguingly, EPHA2, largely reported for its negative impact on many solid tumors’ pathogenesis, seems to play a beneficial role in lung carcinogenesis. Two studies, which jointly included 420 patients with stage I Non-Small-Cell Lung Carcinoma (NSCLC), suggested that high EPHA2 expression was correlated with well-differentiated tumors, reduced smoking status, adenocarcinoma histological type, stage IA tumors, as well as the presence of *Epidermal Growth Factor Receptor (EGFR)* gene mutations. Both groups of researchers indicated EPHA1 as a positive prognostic factor for NSCLC patients [21,22]. EPHA4, EPHA5, and EPHA7 also exert a positive influence on lung cancer tumorigenesis. In the aforementioned study by Giaginis et al, EPHA4 was associated with low tumor stage, decreased presence of inflammation, and favorable OS. Additionally, EPHA5 overexpression was correlated with tumor proliferative capacity and longer OS and EPHA7 overexpression correlated with tumor proliferative capacity and older age, presence of fibrosis, as well as with smaller tumor size [20].

#### 4.1.2. EPHBs

EPHs of the B subgroup appear to overwhelmingly contribute to lung cancer tumorigenesis. EPHB1 expression, measured through IHC and Western blot in 60 NSCLC specimens, was higher in cancer tissues compared with non-neoplastic ones. Additionally, EPHB1 expression was higher in patients with metastatic disease and shorter OS [23]. EPHB2 exhibited elevated expression in cancer NSCLC tissues, being correlated with increased depth of invasion, higher TNM stage, and decreased patient OS [24]. EPHB4 overexpression was linked to low tumor differentiation, high Ki67 index, and presence of LN metastasis in a series of 93 lung adenocarcinoma tissues [25].

As far as ephrins are concerned, few data currently exist linking their action to lung carcinogenesis. Ephrin-A1 was reported to have no impact on clinical outcome in two studies [21,26], while a third study associated its overexpression with reduced smoking status, adenocarcinoma histological type, high level of tumor differentiation, and the presence of *EGFR* gene mutations, attributing to the ligand a positive influence in tumorigenesis [22]. Ephrin-B2 expression was absent in all 93 lung adenocarcinoma tissues in the aforementioned study [25], and ephrin-B3 expression, while observed as higher in non-squamous tumors, exhibited no effect on patient OS [21]. Table 2 and Figure 3 include information regarding the clinical impact of EPH/ephrin expression in lung cancer.

### 4.2. Breast

The established, immense impact of breast tumors’ molecular signature in disease prognosis and treatment regimens has shifted the focus of the scientific community on those molecular mechanisms implicated in the disease pathogenesis and the search for novel biomarkers that influence the clinical outcome. Among them, the expression and effect of the EPH/ephrin system in breast cancer tumorigenesis has been extensively studied, with overexpression of all members contributing to worse disease prognosis [27,28,29,30,31,32,33,34].

#### 4.2.1. EPHAs

EPHA2 expression has been reported as higher in cancer tissues compared with non-cancerous epithelium, elevated EPHA2 expression being associated with reduced OS, while EPHA2 and ephrin-A1 co-expression are correlated with recurrence rates [28]. EPHA4 overexpression was also associated with malignancy and cases with reduced OS [28]. In addition, data mining from the GOBO and ONCOMINE databases indicated that elevated EPHA4 *mRNA* expression correlated with stage 3 and 4 tumors, poor tumor differentiation, shortened relapse-free survival, positive LN status, and the highly aggressive basal-like subtype [29]. EPHA7 exhibited higher expression in cancer tissues compared with normal ones and in samples from patients with reduced OS as well [28], while EPHA8 expression exhibited no significant increase in cancerous tissues compared with normal ones and did not affect clinical outcomes [28].

#### 4.2.2. EPHBs

EPHB1 expression showed no correlation with clinical parameters [30]. On the other hand, the varying impact of EPHB2 expression in breast cancer patients’ clinical outcomes reflects the complex role of EPH/ephrin molecular mechanisms in tumorigenesis. EPHB2 exhibited both membranous and cytoplasmic staining in IHC, showing an inverse correlation between the two patterns. Cytoplasmic expression of EPHB2 negatively affected patients’ outcomes, as it was positively associated with shorter OS, histological grade, and HER2 expression. On the contrary, membranous EPHB2 staining was correlated with longer patient OS [27]. Other studies yielded contradictory results regarding the influence of EPHB2 expression in breast cancer pathogenesis, with Wu et al linking its overexpression to decreased OS and DFS [31], and another group of researchers associating it with improved patient prognosis [30]. Mu et al associated elevated mRNA EPHB3 expression with worse OS and EPHB4 mRNA overexpression with improved RFS [30]. In contrast, other studies underlined that EPHB4 overexpression is correlated with malignancy and reduced OS [28], higher histological grade and stage [31], as well as with shorter metastasis-free survival [32]. Despite its beneficial influence in many neoplastic diseases, EPHB6 seems to promote tumorigenesis in the breast, as its expression was reported as higher in malignant tissues and was correlated with reduced OS [28].

#### 4.2.3. Ephrins

Ephrin-A1 and EPHA2 co-expression is associated with recurrence [27]. Similarly, ephrin-B2 expression is associated with the clinically aggressive basal-like breast cancer subtype and HER2(+) tumors [33], while its overexpression is associated with the presence of LN metastasis, HER2 positivity, and the triple-negative breast cancer subtype [34]. On the other hand, the protective role of ephrin-B2 was underlined through the association of its expression with high estrogen receptor expression, low HER2 expression, low histological grade, and good patient’ prognosis [32].

All information retrieved on the clinical impact EPH/ephrin system expression is presented in Table 2 and Figure 3.

### 4.3. Skin

EPHA1 expression in 56 basal cell and 32 squamous cell carcinoma was examined through IHC and q RT-PCR and compared to 10 normal skin tissues. Cancer tissues exhibited downregulation of EPHA1 in relation to normal tissues, and EPHA1 decreased expression associated with increased tumor thickness in squamous cell carcinoma cases [35].

On the other hand, EPHA2 overexpression in melanoma tissues was correlated with increased melanoma thickness and increased tumor cell proliferative capacity, assessed as the Ki67 index. In addition, high expression of its ligand, ephrin-A1, was also correlated with increased melanoma thickness, as well as decreased patient survival [36].

Data from the two studies on EPH/ephrins and skin cancer are presented in Table 2 and Figure 3.

**Table 2:** EPHs/ephrins (bold) studied in solid tumors of lung, breast, and skin and correlations with clinicopathological parameters.

EPHs/Ephrins	Malignant Tissues	Benign Control Tissues	Methods	Results	Refs
**LUNG**					
**EPHA1**	88 NSCLC (56 lung adenocarcinoma, 32 squamous cell carcinoma)		TMA IHC	**EPHA1** expression associated with tumor-proliferative capacity	[20]
**EPHA2**	225 NSCLC node-negative stage I samples		TMA IHC	**EPHA2** expression reported: ○ higher in well-differentiated tumors ○ to be a positive prognostic factor, associated with improved OS	[21]
	195 stage I NSCLC samples		Real-time RT-PCR	High **EPHA2** expression correlated with ○ female sex ○ reduced smoking status ○ adenocarcinoma ○ well-differentiated carcinomas ○ p-stage IA tumors ○ EGFR gene mutations High **EPHA2** expression linked to improved DFS	[22]
**EPHA4**	88 NSCLC (56 lung adenocarcinoma, 32 squamous cell carcinoma)		TMA IHC	**EPHA4** expression associated with increased OS **EPHA4** expression associated with low tumor stage and presence of inflammation	[20]
**EPHA5**	88 NSCLC (56 lung adenocarcinoma, 32 squamous cell carcinoma)		TMA IHC	**EPHA5** expression associated with tumor-proliferative capacity **EPHA5** associated with increased OS	[20]
**EPHA7**	88 NSCLC (56 lung adenocarcinoma, 32 squamous cell carcinoma)		TMA IHC	**EPHA7** expression associated with tumor-proliferative capacity **EPHA7** expression associated with older age, fibrosis and smaller tumor size	[20]
**EPHB1**	60 NSCLC samples	Non-cancerous tissues	Western blot, IHC	Increase in **EPHB1** expression detected in NSCLC tissues compared with non-cancerous ones Increased **EPHB1** expression reported in patients with metastasis High **EPHB1** expression correlated with poorer OS	[23]
**EPHB2**	126 lung adenocarcinoma samples	corresponding non-cancerous lung tissues	Quantum dots IHC	**EPHB2** expression higher in cancer tissues • High **EPHB2** expression correlated with ○ increased depth of invasion ○ higher TNM stage ○ decreased OS	[24]
**EPHB4**	93 lung adenocarcinoma samples		IHC	**EPHB4** differentially expressed in lung adenocarcinoma **EPHB4** expression linked to ○ low tumor differentiation ○ high Ki67 expression ○ LN metastasis No correlation between **EPHB4** expression and sex, age, or ALK mutation	[25]
**ephrin-A1**	225 NSCLC node-negative stage I samples		TMA IHC	**ephrin-A1** expression had no impact on survival	[21]
	195 stage I NSCLC samples		Real-time RT-PCR	High **ephrin-A1** expression correlated with ○ female gender ○ reduced smoking status ○ adenocarcinoma ○ well-differentiated carcinomas ○ p-stage IA tumors ○ EGFR gene mutations	[22]
	244 NSCLC samples		IHC	No association between **ephrin-A1** expression and clinical outcome	[26]
**ephrin-B2**	93 lung adenocarcinoma samples		IHC	**ephrin-B2** negative in all tissues	[25]
**ephrin-B3**	225 NSCLC node-negative stage I samples		TMA IHC	**ephrin-B3** expression ○ higher in non-squamous tumors ○ had no effect on patient OS	[21]
**BREAST**					
**EPHA2**	mRNA expression and patients’ survival data from 412 patients from databases		mRNA and survival data analysis	**EPHA2** expression correlated with reduced OS **EPHA2** and **ephrin-A1** co-expression associated with disease recurrence **EPHA2** expression higher in tumor tissues than normal ones	[28]
	TMA IHC in 126 invasive ductal adenocarcinoma samples	8 normal/benign epithelium samples	TMA IHC		
**EPHA4**	mRNA expression and patients’ survival data from 412 patients from databases		mRNA and survival data analysis	**EPHA4** expression correlated with reduced OS **EPHA4** expression higher in tumor tissues than normal ones	[28]
	TMA IHC in 126 invasive ductal adenocarcinoma samples	8 normal/benign epithelium samples	TMA IHC		
	No number mentioned		GOBO and ONCOMINE databases used to investigate EPHA4 mRNA expression and clinical correlations	High ***EPHA4*** *mRNA* expression correlated with ○ the highly aggressive basal-like subtype ○ stage 3 and 4 tumors ○ poorly differentiated (stage 3) tumors ○ shortened relapse-free survival ○ positive LN status	[29]
**EPHA7**	mRNA expression and patients’ survival data from 412 patients from databases		mRNA and survival data analysis	**EPHA7** expression correlated with reduced OS **EPHA7** expression higher in tumor tissues than normal ones	[28]
	TMA IHC in 126 invasive ductal adenocarcinoma samples	8 normal/benign epithelium samples	TMA IHC		
**EPHA8**	mRNA expression and patients’ survival data from 412 patients from databases		mRNA and survival data analysis	**EPHA8** not associated with clinical outcome	[28]
	TMA IHC in 126 invasive ductal adenocarcinoma samples	8 normal/benign epithelium samples	TMA IHC		
**EPHB1**	3554 breast cancer samples		*mRNA* expression data and survival information from the KM plotter database	High *mRNA expression* of all EPHs studies associated with increased risk of mortality for LN (+) patients **EPHB1** expression not associated with OS	[30]
**EPHB2**	Cohort 1: 65 LN positive breast cancer samples		RT-PCR	Inverse correlation between membranous and cytoplasmic **EPHB2** expression Membranous **EPHB2** expression correlated with longer OS, while cytoplasmic **EPHB2** expression with shorter OS Cytoplasmic **EPHB2** expression positively correlated with histological grade and HER2 expression	[27]
	Cohort 2: 216 breast cancer samples		IHC		
	3554 breast cancer samples		*mRNA* expression data and survival information from the KM plotter database	High *mRNA expression* of **EPHB2** associated with improved OS High *mRNA expression* of all EPHs studied associated with increased risk of mortality for LN (+) patients	[30]
	94 breast carcinoma samples	9 normal breast tissue samples	IHC Semi-quantitative RT-PCR	Increased **EPHB2** expression negatively associated with OS and DFS	[31]
**EPHB3**	3554 breast cancer samples		*mRNA* expression data and survival information from the KM plotter database	High mRNA expression of **EPHB3** associated with worse OS High mRNA expression of all EPHs studies associated with increased risk of mortality for LN (+) patients	[30]
**EPHB4**	mRNA expression and patients’ survival data from 412 patients from databases		mRNA and survival data analysis	**EPHB4** expression correlated with reduced OS **EPHB4** expression higher in tumor tissues than normal ones	[28]
	TMA IHC in 126 invasive ductal adenocarcinoma samples	8 normal/benign epithelium samples	TMA IHC		
	3554 breast cancer samples		*mRNA* expression data and survival information from the KM plotter database	High mRNA expression of **EPHB4** associated with improved OS High mRNA expression of all EPHs studied associated with increased risk of mortality for LN (+) patients	[30]
	94 breast carcinoma samples	9 normal breast tissue samples	IHC Semi-quantitative RT-PCR	**EPHB4** expression associated with higher histological grade and stage	[31]
	216 breast cancer samples		IHC	High **EPHB4** expression associated with shorter metastasis-free survival	[32]
**EPHB6**	mRNA expression and patients’ survival data from 412 patients from databases		mRNA and survival data analysis	**EPHB6** expression correlated with reduced OS **EPHB6** expression higher in tumor tissues than normal ones	[28]
	TMA IHC in 126 invasive ductal adenocarcinoma samples	8 normal/benign epithelium samples	TMA IHC		
**ephrin-A1**	mRNA expression and patients’ survival data from 412 patients from databases		mRNA and survival data analysis	**EPHA2** and **ephrin-A1** co-expression associated with recurrence	[28]
	TMA IHC in 126 invasive ductal adenocarcinoma samples	8 normal/benign epithelium samples	TMA IHC		
**ephrin-B1**	96 samples		Gene expression data from SAGA and MicMa databases	**ephrin-B1** expression associated with basal-like breast cancer subtype and HER2(+) breast cancer	[33]
	75 breast cancer samples (71 ductal adenocarcinoma, 4 lobular adenocarcinoma)		IHC	High **ephrin-B1** expression correlated with ○ LN metastasis ○ presence of HER2 receptor ○ triple—negative breast carcinoma subtype	[34]
**ephrin-B2**	216 breast cancer samples		IHC	High **ephrin-B2** expression correlated with ○ high ER expression ○ low HER2 expression ○ low histological grade ○ longer OS	[32]
**SKIN**					
**EPHA1**	56 basal cell carcinomas, 32 squamous cell carcinomas	10 normal skin samples	IHC qRT-PCR	**EPHA1** downregulated in cancer tissues compared with normal skin **EPHA1** downregulation associated with increased tumor thickness, in squamous cell carcinoma	[35]
**EPHA2**	202 vertical growth phase melanomas 68 separate samples of local (skin), regional (LNs) and distant metastasis from 58 patients with recurrent disease		TMA IHC	**EPHA2** overexpression associated with: ○ increased melanoma thickness ○ increased tumor cell proliferation capacity (high Ki67 expression)	[36]
**ephrin-A1**	202 vertical growth phase melanomas 68 separate samples of local (skin), regional (LNs) and distant metastasis from 58 patients with recurrent disease		TMA IHC	**ephrin-A1** overexpression associated with: ○ increased melanoma thickness ○ decreased OS	[36]

NSCLC: non-small cell lung carcinoma, TMA: tissue microarrays, IHC: immunohistochemistry, LN: lymph nodes, RT-PCR: reverse transcription-polymerase chain reaction, EGFR: epidermal growth factor receptor, DFS: disease-free survival, ALK: anaplastic lymphoma kinase, OS: overall survival.

## 5. Gastrointestinal Tract Neoplasia

### 5.1. Esophagus

**Figure 4:**
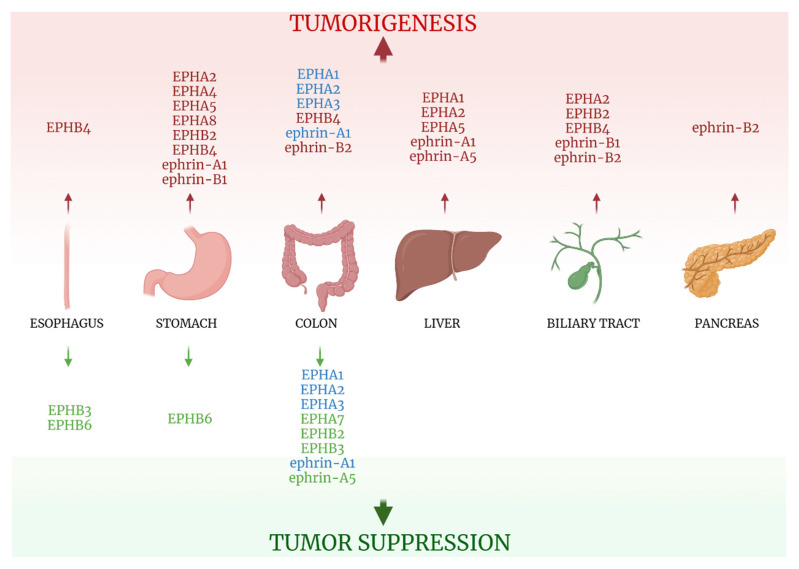
EPH/ephrin family role in solid tumors of organs of the gastrointestinal tract, liver, biliary tract, and pancreas. Red-colored EPHs/ephrins enhance tumorigenesis, while green-colored ones suppress it. It is unclear if EPHA1, EPHA2, EPHA3, and ephrin-A5 enhance tumorigenesis or tumor suppression (blue font). See text and Table 3. Created with BioRender.com (accessed on 2 August 2021).

A limited amount of information exists concerning the clinical impact of the EPH/ephrin system expression in esophageal carcinomas. Schauer et al investigated the expression of EPHB3 and E-cadherin in healthy esophagi and Barrett’s carcinoma patients, reporting that both were reduced in adenocarcinoma tissues compared with normal or dysplastic ones [37]. Simultaneous expression of the two proteins showed an inverse correlation with tumor stage. Interestingly, when EPHB3 was expressed, redistribution of E-cadherin from the cytoplasm to the membrane was observed, revealing a possible mechanism through which EPHB3 might exert its speculated protective role, as E-cadherin membranous expression stabilizes cell to cell adhesion, decreasing tumor cells’ invasion capabilities, and disrupts EMT transition and cell proliferation. Other studies reported that EPHB4 was gradually overexpressed, from preneoplastic lesions to gastroesophageal cancers, and was correlated positively with advanced tumor stage. On the other hand, EPHB6 expression was down-regulated in neoplastic tissues, in accordance with its tumor-suppressive properties observed in other cancers [38]. Information concerning the clinical impact of the EPH/ephrin system expression in esophageal cancers is summarized in Table 3 and Figure 4.

### 5.2. Stomach

#### 5.2.1. EPHAs/Ephrin-As

The clinical impact of EPH/ephrin member proteins’ expression in gastric cancer is among the most extensively studied. Most researchers utilized a variety of methods, such as IHC, western blot, or RT-PCR, to assess EPH/ephrin protein or mRNA expression and proceeded to correlate it with clinicopathological parameters.

Yuan et al [39] studied the expression of EPHA2 and ephrin-A1 in 176 gastric adenocarcinoma specimens, underlining that they were significantly overexpressed in cancer tissues when compared with normal ones. Expression of both proteins was correlated with increased TNM stage and LN metastasis rate, with EPHA2 overexpression also linked to deeper levels of tumor invasion and found to be an independent poor prognostic factor, as its increased expression correlated with decreased OS. Nakama et al, however, observed that while *EPHA2*/*ephrin-A1* genes expression was associated with tumors that invaded in deeper layers of the stomach and had undefined borders or infiltrated diffusely, no correlation existed regarding the tumor size, patients’ age, vessel infiltration, and lymph node status [40]. On the contrary, in a large study that included 91 gastric adenocarcinoma samples, high EPHA2 protein expression was associated with advanced tumor stage, size, LN metastasis, TNM stage, and lymphovascular invasion [41].

EPHA4 and EPHA8 also seem to hold tumorigenic properties. Both proteins’ expression was linked to depth of malignant infiltration of the stomach and lower OS [42,43], with high EPHA8 expression additionally accompanied by lower tumor differentiation, as well as higher TNM stages and distant metastasis rate.

Interestingly, overexpression of a number of EPHs, belonging in A and B subgroups, was also observed in gastric stromal cells associated with gastric adenocarcinoma [44]. Such results possibly prove that EPHs exert their carcinogenic properties through their involvement in cell adhesion and matrix-cell interactions, creating a direct clinical impact. Ephrin ligands expressed on tumor cells could possibly help them interact with stromal cells expressing EPHs, promoting carcinogenic properties such as increased cell motility that would enhance tissue infiltration or triggering molecular cascades that promote cell proliferation. The EPH/ephrin system may represent a key factor in the creation of a tumor-promoting microenvironment, and further study regarding their expression in non-tumorous tissues surrounding cancer cells is called for.

#### 5.2.2. EPHBs/Ephrin-Bs

EPHs of the B subgroup and their ligands also seem to play a role in gastric cancer pathogenesis. Ephrin-B1 expression was positively correlated with the tumor differentiation level [45]. EPHB4 expression, that was reported higher in Barrett esophagus, was also recorded elevated in gastroesophageal carcinomas, tumors of higher stage and in malignant cells at the tumor invasion front [38]. On the other hand, two studies underline the protective role of EPHB6, as its expression was found to be downregulated in tumor cells [38], being negatively associated with tumor stage, LN metastasis, and a poor level of differentiation [46].

Lastly, ephrin-B1 expression was reported more frequent in poorly differentiated adenocarcinomas than well-differentiated ones [45].

Data from research on EPH/ephrin expression and clinicopathological features in gastric cancer are presented in Table 3 and Figure 4.

### 5.3. Colon

Colorectal cancer (CRC) is the third most common malignancy in the world and the third leading cause of cancer-related deaths [47]. The heavy clinical impact of the disease has led to extensive research on its pathogenesis, including the tumorigenic role of the EPH/ephrin system. Strikingly, many EPHs and their ligands seem to have a protective function [48,49,50,51,52,53,54,55,56,57,58,59,60].

#### 5.3.1. EPHAs

EPHA1, although increased in CRC tissues compared with normal ones, exhibited decreased expression in advanced TNM disease stages [48,49], with one study correlating its low expression to poor OS [49]. Similarly, EPHA2 was found to be upregulated in CRC tissues compared with their non-cancerous counterparts, but its downregulation was prevalent in more advanced disease stages [48,50]. Moreover, EPHA2 overexpression was observed in tumors less than 5 centimeters in diameter compared with larger ones [50]. EPHA3 and EPHA7 exhibited loss of expression in 96% of the cases in one of the three previous studies [48]. On the contrary, Li et al [51] reported that EPHA3 not only was overexpressed in CRC tissues compared with adjacent normal mucosa, but was also positively linked to patients’ age, poor differentiation level of the tumor, and LN metastasis.

#### 5.3.2. EPHBs

The inhibitory role of EPHB2 in colon carcinogenesis is among the ones most thoroughly studied. Multiple works have investigated its clinical impact, associating its high expression levels with lower tumor stage [54,57], lower histological grade [58], higher grade of histological differentiation [54], low LN metastasis rate [57], longer OS and DFS [54,56,57], and lower recurrence and death rates [55]. In a large series of tissue specimens from CRC patients, scientists have demonstrated that from normal colon mucosa to preneoplastic lesions, to malignant tissues, to the tumor invasion front, to metastasis, EPHB4 expression is progressively reduced [54,57,58].

Jan et al examined the expression of EPHB3 in a total of 642 specimens, reporting its attenuation during transition from adenoma to carcinoma and its further decline as the tumor invaded into deeper tissues. Statistical analysis linked EPHB3 expression to tumor differentiation, lymphovascular invasion, TNM stage, microsatellite instability and better OS [59].

In contrast, EPHB4 was presented in one study as being among the few members of the EPH/ephrin family that favor tumorigenesis. While its expression was minimal to absent in normal colon tissues, it was strongly expressed in CRC and associated with tumor depth of invasion, presence of LN and distant metastasis and TNM stage [60].

#### 5.3.3. Ephrins

Ephrin-A1 expression, although higher in CRC tissues compared with normal ones [48,50], was correlated to early-stage cancers and smaller tumor size [50]. Ephrin-A5 expression, albeit found higher than its EPHA receptors [48], was significantly reduced in colon cancer tissues and was negatively associated with tumor differentiation and clinical stage [52]. Ephrin-B2 expression, contrary to the seemingly tumor suppressive properties of ephrin-A5, was found elevated in CRC samples, with frequent enhancement on the luminal surface of carcinoma epithelium [53]. All information on EPH/ephrin expression on CRC tissues is summarized in Table 3 and Figure 4.

### 5.4. Liver

All of the EPH/ephrins investigated in Hepatocellular Carcinoma (HCC), with the only exception of ephrin-A5, seem to have a negative impact on carcinogenesis and patients’ clinical outcomes. Ephrin-A1 expression was reported as lowest in normal liver tissues, elevated in cirrhotic liver specimens, and further enhanced in HCC [61,62]. Its expression was correlated with Alpha Fetoprotein expression, which was associated with poor OS [61]. Wada et al also linked ephrin-A1 expression to poorer patients’ prognosis [63]. EPHA1 exhibited strong expression in HCC specimens [61], and EPHA2 expression was associated with decreased differentiation and shorter OS [62], as well as with microscopic portal invasion. A study that examined the expression of three kinases (Anaplastic Lymphoma Kinase, Fibroblast Growth Factor Receptor 2, and EPHA5) in a series that included 250 HCC cases, underlined that coactivation of all three of them led to a worse prognosis [64]. On the contrary, Wang et al [65] examined the mRNA expression of the two alternative isoforms of ephrin-A5, large ephrin-A5 (ephrin-A5L), and small ephrin-A5 (ephrin-A5S), through means of quantitative real-time PCR, reporting them lower in HCC compared with peritumoral tissues. Ephrin-A5S expression positively correlated with old age and histological grade, and its expression in peritumoral tissues was associated with better DFS and OS. Data are presented in Table 3 and Figure 4.

### 5.5. Biliary Tract

Limited data is currently available regarding the role of EPHs and their ligands in biliary tract neoplasia and its clinical significance. Sheng et al observed that the EPHA2 gene was frequently mutated in primary intrahepatic cholangiocarcinoma (ICC) tumors, with mutations even more likely to occur in cases with lymph node metastasis [66]. Another study included 50 ICC patients to examine EPHB2, EPHB4, ephrin-B1, and ephrin-B2 expression, as well as tumors’ MVD through CD34 IHC staining [67]. An association between metastasis status and EPHB2 expression, EPHB2/ephrin-B1 co-expression, EPHB2/ephrin-B2 co-expression, EPHB4 expression along with high MVD and lastly ephrin-B2 expression and high MVD, was reported.

Information regarding the EPH/ ephrin system’s role in biliary tract neoplasia is summarized on Table 3 and Figure 4.

### 5.6. Pancreas

The only study examining the clinical significance of EPHs/ephrins members expression in pancreatic cancer is the one conducted by Zhu et al [68] and concerned the *ephrin-B2* gene expression in pancreatic ductal adenocarcinoma (PDAC). The researchers gathered information on ephrin-B2 mRNA expression from 179 PDAC patients and 171 normal pancreatic tissues. Statistical analysis showed that ephrin-B2 expression was higher in PDAC tissues when compared with normal ones and correlated with shorter OS and DFS. Furthermore, samples from 54 PDAC tissues and adjacent uninvolved tissues were tested for ephrin-B2 expression through IHC, western blot, and q real-time PCR analysis. Ephrin-B2 expression was again reported higher in cancerous tissues, and its overexpression was linked to tumor TNM stage and high Ki67 expression. Table 3 and Figure 4 summarize the study.

**Table 3:** EPHs/ephrins (bold) studied in solid tumors of the gastrointestinal tract, liver, biliary tract, and pancreas and correlations with clinicopathological parameters.

EPHs/Ephrins	Malignant Tissues	Benign Control Tissues	Methods	Results	Refs
**ESOPHAGUS**					
**EPHB3**	141 Barrett’s carcinoma samples	20 healthy esophagi samples	IHC q PCR	**EPHB3** and E-cadherin IHC expression was reduced in adenocarcinoma compared with dysplasia or healthy esophageal mucosa Simultaneous expression of E-cadherin and **EPHB3** showed inverse correlation with the tumor stage E-cadherin mRNA expression reduced in adenocarcinoma compared with dysplasia	[37]
**EPHB4**	31 samples (GEJ and gastric carcinomas) from 30 patients (1 patient had 1 GEJ carcinoma and 1 gastric cancer in stomach corpus)	Paired normal samples	IHC q RT-PCR	**EPHB4** overexpressed in: ○ preneoplastic gastroesophageal lesions ○ furtherly overexpressed in gastroesophageal cancers ○ advanced tumor stage ○ overexpression at the tumor invasion front and vascular endothelial cells	[38]
**EPHB6**	31 samples (GEJ and gastric carcinomas) from 30 patients	Paired normal samples	IHC q RT-PCR	**EPHB6** was down-regulated, consistent with its tumor-suppressive properties in other cancers	[38]
**STOMACH**					
**EPHA2**	176 gastric adenocarcinoma samples	Paired adjacent normal tissues	Real-time RT-PCR IHC Western blot	**EPHA2** expression correlated with: ○ depth of tumor invasion ○ LN metastasis ○ TNM stage **EPHA2** is an independent poor prognostic factor, and its overexpression is linked to poor OS	[39]
	46 adenocarcinomas, 2 adenosquamous carcinomas, 1 neuroendocrine carcinoma	Corresponding non-tumor samples	Semiquantitative RT-PCR IHC Western blot	**EPHA2** expression linked to tumor invasion and tumors with undefined borders or that infiltrated diffusely No correlation between **EPHA2** expression and tumor size/age/vessel infiltration/LN metastasis	[40]
	91 gastric adenocarcinomas	7 gastrointestinal stromal tumor samples	IHC Western blot	High **EPHA2** expression correlated with: ○ advanced stage ○ tumor size ○ LN metastasis ○ lymphovascular invasion ○ TNM stage	[41]
	107 gastric adenocarcinoma samples (54 of them received adjuvant chemotherapy)	Normal paired gastric samples	Proteome analysis (LC-MS/MS) mRNA (real-time RT-PCR) IHC	**EPHA2** expression was 2-fold higher in GCSC than GSC Cases that showed intermingling of **EPHA2** (+) cells and GCSCs showed ○ more frequent relapse ○ shorter relapse-free survival	[44]
**EPHA4**	24 paired fresh gastric adenocarcinoma samples, 74 fresh frozen paraffin embedded gastric adenocarcinoma samples, 55 gastric adenocarcinoma samples in tissue microarrays	Adjacent non-tumor samples from 24 gastric adenocarcinoma specimens	Real-time RT-PCR RT-PCR IHC	Overexpression of **EPHA4** in IHC was observed in cancer tissues **EPHA4** protein levels associated with: ○ depth of invasion ○ recurrence **EPHA4** (+) cancer tissues showed shorter OS than **EPHA4** (-) cancers	[42]
**EPHA5**	107 gastric adenocarcinoma samples (54 of them received adjuvant chemotherapy)	Normal paired gastric samples	Proteome analysis (LC-MS/MS) mRNA (real-time RT-PCR) IHC	**EPHA5** expression was 2-fold higher in GCSC than GSC	[44]
**EPHA8**	206 gastric cancer samples	32 normal gastric mucosa, 60 paracancerous samples	IHC Western blot	**EPHA8** expression associated with: ○ differentiation level ○ TNM stage ○ depth of infiltration ○ distant metastasis ○ poor OS	[43]
**EPHB2**	107 gastric adenocarcinoma samples (54 of them received adjuvant chemotherapy)	Normal paired gastric samples	Proteome analysis (LC-MS/MS) mRNA (real-time RT-PCR) IHC	**EPHB2** expression was 2-fold higher in GCSC than GSC	[44]
**EPHB4**	107 gastric adenocarcinoma samples (54 of them received adjuvant chemotherapy)	Normal paired gastric samples	Proteome analysis (LC-MS/MS) mRNA (real-time RT-PCR) IHC	**EPHB4** expression was 2-fold higher in GCSC than GSC	[44]
	31 gastric and GEJ carcinomas	Paired normal samples	Quantitative real-time RT-PCR IHC	**EPHB4** expression: ○ high in Barrett esophagus ○ high in gastroesophageal cancers ○ associated with advanced tumor stages ○ high at the tumor invasion front	[38]
**EPHB6**	31 gastric and GEJ carcinomas	Paired normal samples	Quantitative real-time RT-PCR IHC	**EPHB6** downregulated in tumor tissues	[38]
	152 gastric carcinoma samples		IHC	**EPHB6** expression: Positively associated with tumor differentiation Negatively associated with ○ LN metastasis ○ tumor stage ○ female sex Showed no association with age/tumor location/depth of invasion	[46]
**ephrin-A1**	176 gastric adenocarcinoma samples	Paired adjacent normal samples	Real-time RT-PCR IHC Western blot	**ephrinA1** expression correlated with: ○ TNM stage ○ LN metastasis	[39]
	46 adenocarcinomas, 2 adenosquamous carcinomas, 1 neuroendocrine carcinoma	Paired non-tumor samples	Semiquantitative RT-PCR IHC Western blot	No correlation between **ephrin-A1** expression and tumor size/age/vessel infiltration/LN metastasis	[40]
**ephrin-B1**	29 gastric carcinoma samples	Matched normal samples	Semiquantitative RT-PCR	**ephrin-B1** expression more frequent in poorly differentiated adenocarcinomas than well-differentiated ones	[45]
**COLON**					
**EPHA1**	53 CRC samples	adjacent normal samples	IHC q real-time PCR	**EPHA1** expression increased in CRC tissues compared with normal ones **EPHA1** downregulation was prevalent in more advanced TNM stages	[48]
	125 CRC specimens, 53 paired normal colon-CRC samples	18 normal colon samples, paired normal samples from the 53 CRC patients	q real-time PCR Flow cytometry Western blot IHC	**EPHA1** upregulated in 50% of the cases **EPHA1** overexpression more prevalent in stage 2 compared with stage 3 CRC Low **EPHA1** expression correlated with poor OS	[49]
**EPHA2**	53 CRC samples	adjacent normal samples	IHC q real-time PCR	**EPHA2** expression increased in CRC tissues compared with normal ones **EPHA2** downregulation was prevalent in more advance TNM stages	[48]
	37 CRC samples	37 paired normal samples	IHC Semi-quantitative RT-PCR	**EPHA2** overexpressed: ○ in tumor tissues compared to adjacent normal tissue ○ early stage cancers compared to late stage cancers ○ in smaller tumors (<5 cm)	[50]
**EPHA3**	53 CRC samples	adjacent normal tissues	IHC q real-time PCR	**EPHA3** showed loss of expression in 96% of cases	[48]
	68 CRC samples	paired adjacent normal mucosa	IHC	**EPHA3** highly expressed in CRC tissues compared with normal mucosa **EPHA3** expression level associated with: ○ age ○ tumor differentiation ○ LN metastasis	[51]
**EPHA7**	53 CRC samples	adjacent normal samples	IHC q real-time PCR	**EPHA7** showed loss of expression in 96% of cases	[48]
**EPHB2**	345 primary CRCs 98 LN metastasis 82 liver metastasis	100 adenomas 111 normal mucosa	IHC	**EPHB2** expressed in: ○ 100% normal colon crypt base cells ○ 78% adenomas ○ 55.4% CRCs ○ 37.8% LN metastasis ○ 32.9% liver metastasis **EPHB2** expression negatively correlated with: ○ tumor stage ○ poor differentiation ○ poor OS ○ poor DFS From tumor to metastasis, CRC carcinogenesis shows a progressive loss of **EPHB2** expression	[54]
	159 samples of stage 3 CRC		IHC	Decrease of **EPHB2** expression correlated independently with recurrence	[55]
	28 primary CRCs 39 metastasis 342 paired primary CRCs and normal samples in tissue microarrays	28 normal colon samples 148 colorectal adenomas	IHC	**EPHB2** expressed in normal crypts, colorectal adenomas, primary cancers, and metastasis High **EPHB2** expression associated with longer OS in CRC	[56]
	4000 samples from 138 different tumor types, 1476 samples of colon cancer	76 different normal samples	IHC	**EPHB2** expression predominantly found in intestinal epithelium Loss of **EPHB2** expression associated with: ○ high pT tumor stage ○ nodal positivity ○ infiltrative tumor margin ○ shorter OS	[57]
	36 CRCs	30 Dysplastic aberrant foci, (dACF) 31 small (<5 mm) adenomas, 12 large (>5 mm) adenomas	IHC	100% of dACFs and 61% of small adenomas retained **EPHB2** expression All CRCs showed extensive loss of **EPHB2** expression (>50%) and 25% of CRCs were entirely negative **EPHB2** downregulation associated with higher histological grade	[58]
**EPHB3**	610 CRC samples formalin-fixed 32 fresh colorectal cancer samples		IHC q real-time PCR	**EPHB3** higher in CRC tissues than in normal mucosa **EPHB3** declined during transformation from adenoma to carcinoma and as the tumor invaded into deeper tissues Budding cancer cells at the invasive tumor fronts showed reduction of **EPHB3** expression **EPHB3** expressed in 24% of 610 CRCs **EPHB3** expression negatively associated with: ○ tumor differentiation ○ lympho-vascular invasion ○ TNM stage **EPHB3** expression positively associated with MSI **EPHB3** expression correlated with better clinical outcomes, but was not and independent prognostic marker	[59]
**EPHB4**	20 colon carcinoma samples	adjacent uninvolved mucosa	IHC RT-PCR	**EPHB4** frequently expressed on the luminal surface	[53]
	200 CRC samples	50 adjacent non-tumor samples	IHC	**EPHB4** strongly expressed in CRC cells but minimal to no expression in normal colon mucosa **EPHB4** associated with: ○ invasion depth ○ LN metastasis ○ distant metastasis ○ TNM stage	[60]
**ephrin-A1**	53 CRC samples	adjacent normal samples	IHC q real-time PCR	**Ephrin-A1** expression increased in CRC compared with normal tissue in 51% of cases	[48]
	37 CRC samples	37 paired normal samples	IHC Semi-quantitative RT-PCR	**Ephrin-A1** overexpressed: ○ in tumor tissues compared with adjacent normal tissue ○ early stage cancers compared with late stage cancers ○ in smaller tumors (<5 cm)	[50]
**ephrin-A5**	53 CRC samples	adjacent normal samples	IHC Quantitative real-time PCR	**EphrinA5** expression higher in CRC tissues compared to its high affinity receptors	[48]
	72 colon malignancies (68 adenocarcinomas, 4 non-Hodgkin lymphomas)	10 normal colon samples 14 benign colon lesions (adenomas and polyps)	IHC Western blot q real-time PCR RT-PCR	**EphrinA5** mRNA and protein levels significantly reduced in colon cancer compared to normal colon **EphrinA5** negatively associated with: ○ tumor differentiation ○ clinical stage	[52]
**ephrin-B2**	20 colon carcinoma samples	adjacent uninvolved mucosa	IHC RT-PCR	**Ephrin-B2** higher expression in CRC than normal mucosa **Ephrin-B2** frequently expressed on the luminal surface	[53]
	200 CRC samples	50 adjacent non-tumor samples	IHC	**EphrinB2** expression was the same in CRC and normal tissues	[60]
**LIVER**					
**EPHA1**	20 HCC samples	Non-cancerous adjacent tissues	IHC Northern blot	**EPHA1** and AFP strongly expressed in cancerous tissues	[61]
**EPHA2**	139 HCC samples		Real-time q RT-PCR	**EPHA2** expression correlated with microscopic portal invasion	[63]
	40 HCC samples	Cirrhotic non-tumorous samples	IHC Northern blot RT-PCR	**EPHA2** expression correlated with: ○ decreased differentiation ○ poor OS	[62]
**EPHA5**	250 HCC samples	Paired normal samples	IHC	None of the kinases expression examined (ALK, FGFR, and **EPHA5**) individually showed any correlation with OS, but co-activation of all three kinases showed a worse prognosis	[64]
**ephrin-A1**	20 HCC samples	Non-cancerous adjacent samples	IHC Northern blot	**Ephrin-A1** expression ○ lowest in normal liver tissues ○ elevated in cirrhotic tissues ○ further elevated in HCC **Ephrin-A1** expression strongly correlates with AFP expression, which is associated with poor OS **Ephrin-A1**, and AFP strongly expressed in cancerous tissues	[61]
	139 HCC samples		Real-time q RT-PCR	**Ephrin-A1** expression correlated with decreased DFS	[63]
	40 HCC samples	Cirrhotic non-tumorous samples	IHC Northern blot RT-PCR	**Ephrin-A1** was overexpressed in HCC tissues compared with corresponding non tumor tissues	[62]
**ephrin-A5**	142 paired HCC and peritumoral liver samples	Paired peritumoral samples	Real-time q RT-PCR	**Ephrin-A5s** and **ephrin-A5l** down-regulated in HCC compared with peritumoral tissues **Ephrin-A5s** positively correlated with: ○ old age ○ histological grade **Ephrin-A1s** expression in peritumoral tissue had better DFS and OS	[65]
**BILIARY TRACT**					
**EPHA2**	30 ICC samples, 5 LN metastasis from 5 of the above patients	Normal adjacent samples	PCR-based Sanger sequencing	**EPHA2** frequently mutated in primary ICC tumors **EPHA2** mutations more likely to occur in ICC with LN metastasis	[66]
**EPHB2**	50 cholangiocarcinoma samples		IHC	High **EPHB2** expression **EPHB2**/**ephrin-B1** co-expression **EPHB2**/**ephrin-B2** co-expression correlated with metastasis status	[67]
**EPHB4**	50 cholangiocarcinoma patients		IHC	High **EPHB4** and high MVD correlated with metastasis status	[67]
**ephrin-B1**	50 cholangiocarcinoma samples		IHC	High **EPHB2**/**ephrin-B1** co-expression **Ephrin-B1** expression and high MVD correlated with metastasis status	[67]
**ephrin-B2**	50 cholangiocarcinoma samples		IHC	High **EPHB2**/**ephrin-B2** co-expression correlated with metastasis status	[67]
**PANCREAS**					
**ephrin-B2**	179 PDAC samples (mRNA data)	171 normal pancreatic samples (mRNA data)	Statistical analysis	Data analysis of mRNA expression of the 179 PDAC and 171 normal pancreatic tissues showed that: **Ephrin-B2** expression was higher in PDAC tissues than normal ones High **ephrin-B2** expression correlated with: ○ shorter OS ○ shorter DFS	[68]
	54 PDAC samples	54 adjacent normal samples	IHC Quantitative real-time PCR Western blot	IHC, qRT-PCR and western blot analysis of the 54 PDAC tissues showed that: **Ephrin-B2** expression was higher in PDAC tissues than normal ones High **ephrin-B2** expression correlated with: ○ TNM staging ○ higher ki67 expression	

q PCR: quantitative polymerase chain reaction, IHC: immunohistochemistry, q RT-PCR: quantitative reverse transcription-polymerase chain reaction, q real time PCR: quantitative real time transcription-polymerase chain reaction, GEJ: gastroesophageal junction, LN: lymph nodes, OS: overall survival, DFS: disease-free survival, MVD: microvessel density, GCSC: gastric cancer tissues stromal cells, GSC: normal gastric tissue stromal cells, ICC: intrahepatic cholangiocarcinoma, PDAC: pancreatic ductal adenocarcinoma.

## 6. Urinary Tract Neoplasia

### 6.1. Kidney

#### 6.1.1. EPHAs

EPHA1 and EPHA2 proteins expression, although lower in cancer tissues compared with matched non-malignant ones, was observed as higher in metastatic lesions than in primary tumor specimens. Moreover, positive staining of both EPHs was associated with aggressive tumor features, while positive EPHA1 staining was also linked to poor patient OS [69]. Wang et al studied the IHC expression of EPHA3 in 68 samples of Clear Cell Renal Cell Carcinoma (CCRCC) and adjacent normal kidney tissues, reporting its tumor suppressive properties. EPHA3 exhibited strong immunostaining in normal renal tubes, negative expression in 72% of the CCRCC cases and decreased intensity of immunostaining in positive malignant cases. Loss of EPHA3 expression was positively correlated with tumor size and TNM stage [70].

#### 6.1.2. EPHBs/Ephrins

**Figure 5:**
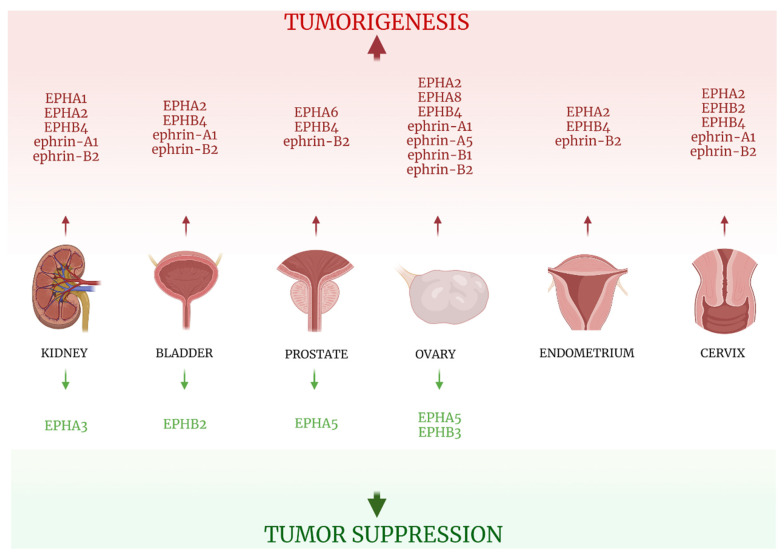
EPH/ephrin family role in solid tumors of organs of the urinary tract, prostate, and gynecological tract. Red-colored EPHs/ephrins enhance tumorigenesis, while green-colored ones suppress it. Created with BioRender.com (accessed on 2 August 2021).

Interestingly, immunohistochemical expression of EPHB4 in the venous endothelium and ephrin-B2 in the arterial endothelium was reported greater in tumoral areas compared with non-tumorous ones [71], possibly underlining the role of the EPH/ephrin system in tumor angiogenesis. Lastly, while ephrin-A1 expression was found to be increased in cancer tissues, its low staining was linked to more aggressive tumor features [69]. All information is presented in Table 4 and Figure 5.

### 6.2. Bladder

#### 6.2.1. EPHAs

The EPH/ephrin system is aberrantly expressed in normal urothelium, with its deregulation implicated in the pathogenesis of transitional cell carcinoma (TCC). EPHA2, similarly to its role in kidney cancer, seems to promote TCC development, as it exhibited low expression in normal urothelial cells and was highly expressed in advancing stages of bladder cancer [72].

#### 6.2.2. EPHBs

TCC tissues showed loss of EPHB2, which is expressed in normal urothelium. On the other hand, TCC specimens exhibited gain of EPHB4 expression, which is absent in normal urothelium, with the signal strength correlating with the highest tumor stage [73]. In addition, EPHB4 expression in venous endothelium was increased in tumor sections compared with benign ones [71].

#### 6.2.3. Ephrins

Ephrin-B2 expression was associated with poor OS and reduced DFS [74] and noted as higher in arterial endothelium of cancer specimens compared with normal ones [71]. Data on EPH/ephrin expression in TCC specimens are presented in Table 4 and Figure 5.

### 6.3. Prostate

#### 6.3.1. EPHAs

Few research data have focused on the role of the EPH/ephrin system in Prostate Cancer (PCa) pathogenesis. EPHA5 is speculated to play a protective role, as loss or downregulation of its expression was reported in 62.2% of PCa specimens but only in 5.1% of Benign Prostate Hyperplasia (BPH) samples. Furthermore, patients with an elevated Gleason score or T3-T4 disease stage exhibited higher rates of *EPHA5* methylation [75]. EPHA6 *mRNA* expression, on the other hand, was elevated in PCa tissues compared with benign ones, and was correlated with vascular and neural invasion, as well as with increased serum levels of prostate-specific antigen and high TNM stage [76].

#### 6.3.2. EPHBs/Ephrin-Bs

In the study conducted by Özgür et al, that enrolled 20 PCa specimens, EPHB4 expression in venous endothelium and ephrin-B2 expression in arterial endothelium was elevated in tumor areas, compared with benign ones [71].

Information concerning EPH/ephrin expression and its clinical impact in prostate cancer is summarized in Table 4 and Figure 5.

**Table 4:** EPHs/ephrins (bold) studied in solid tumors of the urinary tract and prostate and correlations with clinicopathological parameters.

**KIDNEY**					
**EPHs/Ephrins**	**Malignant Tissues**	**Benign Control Tissues**	**Methods**	**Results**	**Refs**
**EPHA1**	mRNA from 75 malignant samples	matched non-malignant samples	q PCR	**EPHA1** expression lower in cancer tissues than normal ones Positive **EPHA1** staining linked to aggressive tumor features Positive **EPHA1** associated with poor OS **EPHA1** expression higher in metastatic than primary lesions Patients with tumors that were: ○ **EPHA1**/ **EPHA2** (+) ○ **EPHA1**/ **ephrin-A1** (+) showed shorter OS	[69]
	protein expression from 241 malignant samples (primary and metastatic)	non-malignant samples	TMA IHC		
**EPHA2**	mRNA from 75 malignant samples	matched non-malignant samples	q PCR	**EPHA2** expression lower in cancer tissues than normal ones Positive **EPHA2** staining linked to aggressive tumor features **EPHA2** expression higher in metastatic than primary lesions Patients with tumors that were: ○ **EPHA1**/ **EPHA2** (+) ○ **EPHA1**/ **ephrin-A1** (+) showed shorter OS	[69]
	protein expression from 241 malignant samples (primary and metastatic)	non-malignant samples	TMA IHC		
**EPHA3**	68 CCRCC samples	adjacent normal kidney samples	IHC	**EPHA3** staining: ○ strong in normal renal tubules ○ decreased in all CCRCC tissues ○ absent in 72% of CCRCC cases Loss of **EPHA3** was associated with: ○ tumor diameter ○ TNM stage No association between **EPHA3** and sex/age/nuclear grade	[70]
**EPHB4**	12 kidney cancer samples		IHC staining of arterial and venous vessels in tumoral and non-tumoral tissues	Expression of **EPHB4** in venous endothelium reported greater in tumoral sections than in non-tumoral ones	[71]
**ephrin-A1**	mRNA from 75 malignant samples	matched non-malignant samples	q PCR	**Ephrin-A1** expression higher in cancer tissues than in normal ones Low **ephrin-A1** staining linked to aggressive tumor features Patients with tumors that were: ○ **EPHA1**/ **EPHA2** (+) ○ **EPHA1**/ **ephrin-A1** (+) showed shorter OS	[69]
	protein expression from 241 malignant samples	non-malignant samples	TMA IHC		
**ephrin-B2**	12 kidney cancer samples		IHC staining of arterial and venous vessels in tumoral and non-tumoral tissues	Expression of **ephrin-B2** in arterial endothelium reported greater in tumoral sections than non-tumoral ones	[71]
**BLADDER**					
**EPHs/Ephrins**	**Malignant Tissues**	**Benign Control Tissues**	**Methods**	**Results**	**Refs**
**EPHA2**	64 TCC samples	13 normal urothelium samples	IHC	**EPHA2** staining intensity lower in normal urothelium and increased greatly in advancing stages of TCC	[72]
**EPHB2**	40 bladder TCC samples	adjacent non-tumorous samples	IHC Western blot	Normal urothelium expresses **EPHB2** TCC samples showed loss of **EPHB2**	[73]
**EPHB4**	40 bladder TCC samples	adjacent non-tumorous samples	IHC Western blot	Normal urothelium does not express **EPHB4** TCC specimens showed gain of **EPHB4** expression **EPHB4** signal strength correlated with highest tumor stage and trended towards the presence of carcinoma in situ	[73]
	33 bladder cancer samples		IHC staining of arterial and venous vessels in tumoral and non-tumoral tissues	Expression of **EPHB4** in venous endothelium reported greater in tumoral sections than non-tumoral ones	[71]
**ephrin-A1**	64 TCC samples	13 normal urothelium samples	IHC	**Ephrin-A1** staining intensity reported low in normal tissues high in cancerous ones, but similar across the various stages of TCC	[72]
**ephrin-B2**	410 bladder TCC samples		Ephrin-B2 expression data from the cancer genome atlas (TCGA)	High **ephrin-B2** expression correlated with: ○ poor OS ○ reduced DFS	[74]
	33 bladder cancer samples		IHC staining of arterial and venous vessels in tumoral and non-tumoral tissues	Expression of **ephrin-B2** in arterial endothelium reported greater in tumoral sections than in non-tumoral ones	[71]
**PROSTATE**					
**EPHs/Ephrins**	**Malignant Tissues**	**Benign Control Tissues**	**Methods**	**Results**	**Refs**
**EPHA5**	22 PCa samples, 23 paired cancerous and non-cancerous samples	39 BPH samples, 23 paired non-cancerous samples	IHC q RT-PCR Methylation-specific PCR	Downregulation or loss of **EPHA5** *mRNA* or protein expression detected in 62.2% PCa tissues and 5.1% BPH tissues Frequency of **EPHA5** DNA methylation was higher in cancer patients with an elevated Gleason score or T3–T4 disease stage	[75]
**EPHA6**	112 PCa tumor samples	58 BPH samples	q real-time PCR	***EPHA6*** mRNA expression reported higher in PCa tumor samples compared with benign specimens **EPHA6** expression correlated with: ○ vascular invasion ○ neural invasion ○ PSA levels ○ TNM stage in PCa	[76]
**EPHB4**	20 PCa samples		IHC staining of arterial and venous vessels in tumoral and non-tumoral tissues	Expression of **EPHB4** in venous endothelium reported greater in tumoral sections than in non-tumoral ones	[71]
**ephrin-B2**	20 PCa samples		IHC staining of arterial and venous vessels in tumoral and non-tumoral tissues	Expression of **ephrin-B2** in arterial endothelium reported greater in tumoral sections than in non-tumoral ones	[71]

q PCR: quantitative polymerase chain reaction, TMA: tissue microarrays, IHC: immunohistochemistry, q RT-PCR: quantitative reverse transcription-polymerase chain reaction, CCRCC: clear cell renal cell carcinoma, TCC: transitional cell carcinoma, OS: overall survival, DFS: disease-free survival, BPH: benign prostate hyperplasia, PCa: prostate carcinoma.

## 7. Gynecological Tumors

### 7.1. Ovary

Gynecological tract neoplasia, along with breast cancer, is greatly impacted by deregulation of the EPH/ephrin system, with its role in ovarian cancer pathogenesis being amongst the most extensively studied.

#### 7.1.1. EPHAs

EPHA1 and EPHA2 expression were reported by Herath et al as having no impact on patients’ survival [77]. However, in a large study that included 118 epithelial ovarian carcinoma (EOC) samples, EPHA2 overexpression was correlated with tumors of higher histological grade and shorter patient OS [78]. EPHA5, in consistency with its tumor suppressive properties on other solid tumors [20,75], exhibits a protective role in ovarian cancer, with its negative or weak expression linked to poor clinical outcomes. Downregulation of EPHA5 seems to contribute to carcinogenesis, since from normal fallopian tissues and benign ovarian neoplasms to borderline tumors and serous carcinomas, a progressive loss of EPHA5 expression was noted [79]. EPHA8 overexpression was reported in EOC tissues and was additionally associated with older age, higher disease stage, positive LNs, presence of distant metastasis, positive ascetic fluid, and high CA-125 levels [80].

#### 7.1.2. EPHBs

EPHB3 also seems to play a beneficial role in ovarian carcinogenesis, with its expression reduced in serous carcinoma tissues compared with benign ones and its downregulation linked to the histological grade and FIGO stage of serous tumors [81]. High EPHB4 expression, on the other hand, was correlated with clinical stage and low patients’ OS [82].

#### 7.1.3. Ephrins

Overexpression in cancer tissues of all of the ligands studied (ephrin-A1, -A5, -B1, and -B2) was linked to adverse clinical outcomes and dismal prognoses.

Ephrin-A1 and ephrin-A5 elevated expression in tumorous specimens was linked to decreased survival [77], while ephrin-B1 overexpression, more frequently observed in high-grade carcinomas, was correlated with an increased recurrence rate and decreased OS [83]. Lastly, ephrin-B2 overexpression in cancer tissues was associated with the clinical stage and low patient OS [82].

Data from studies on the role of the EPH/ephrin system on ovarian cancer clinical outcomes are presented in Table 5 and Figure 5.

### 7.2. Endometrium

Kamat et al reported EPHA2 overexpression by IHC in 48% of 139 endometrial endometrioid carcinoma tissues, but only in 10% of the 10 benign endometrial specimens. In addition, EPHA2 was positively associated with a plethora of adverse clinical characteristics, such as high disease stage, high tumor grade, increased depth of myometrial invasion, low estrogen and progesterone receptors expression, high Ki67 index, and shorter OS [84]. EPHB4 upregulation were correlated with increased clinical stage, tumor dedifferentiation, deeper myometrial invasion, and low OS [85]. Additionally, according to Dong et al, EPHB4 and ephrin-B2 was associated with estrogen receptor expression [86].

Table 5 and Figure 5 summarize the three studies regarding EPHs/ephrins and endometrial carcinoma.

**Table 5:** EPHs/ephrins (bold) studied in solid tumors of the gynecological tract and correlations with clinicopathological parameters.

EPHs/Ephrins	Malignant Tissues	Benign Control Tissues	Methods	Results	Refs
**OVARY**					
**EPHA1**	24 ovarian carcinoma samples	4 benign ovarian samples	q real-time RT-PCR	**EPHA1** expression showed no correlation with OS	[77]
**EPHA2**	24 ovarian carcinoma samples	4 benign ovarian samples	q real-time RT-PCR	**EPHA2** expression showed no correlation with OS	[77]
	118 EOC samples		IHC RT-PCR	**EPHA2** overexpression correlated with higher histological grade High **EPHA2** linked to shorter OS No correlation between **EPHA2** expression and age/histological type/FIGO stage	[78]
**EPHA5**	61 ovarian serous carcinoma samples	24 benign ovarian serous tumors, 42 serous borderline ovarian tumors, 20 normal fallopian tubes	IHC	High **EPHA5** expression reported in: ○ 100% of benign ovarian serous tumors ○ 100% of normal fallopian tubes ○ 76% of serous borderline tumors ○ 31% of ovarian serous carcinomas Negative or weak **EPHA5** expression linked to poor OS	[79]
**EPHA8**	20 fresh-frozen specimens: 20 EOC samples	40 fresh frozen samples: 20 normal ovarian samples, 20 normal fallopian tube samples	q RT-PCR	**EPHA8** *mRNA* and protein expression higher in EOC tissues High **EPHA8** expression associated with older age/higher stage/(+) LNs/metastasis/(+) ascetic fluid/high CA-125 levels	[80]
	125 paraffin-embedded EOCs	98 samples paraffin-embedded: 30 borderline samples, 30 benign ovarian tumors, 20 normal fallopian tubes, 18 normal ovarian samples	TMA IHC		
**EPHB3**	50 ovarian serous carcinomas	17 serous borderline tumors, 19 normal fallopian tubes	IHC	**EPHB3** expression reduced in serous Ca compared with normal fallopian tubes **EPHB3** expression negatively associated with histological grade and FIGO stage of serous carcinomas No correlation between **EPHB3** expr and LN status/tumor involvement of uterus/momentum metastasis/abdomen and pelvic metastasis	[81]
**EPHB4**	72 ovarian cancer samples		IHC Real-time RT-PCR	High **EPHB4** expression correlated with clinical stage and low OS No correlation between **EPHB4** expr levels and histological type	[82]
**ephrin-A1**	24 ovarian carcinoma samples	4 benign ovarian samples	q real-time RT-PCR	**Ephrin-A1** linked to decreased OS	[77]
**ephrin-A5**	24 ovarian carcinoma samples	4 benign ovarian samples	q real-time RT-PCR	**Ephrin-A5** linked to decreased OS **Ephrin-A5**, among the EPHs/ephrins studies, had the dominating influence on survival	[77]
**ephrin-B1**	27 serous adenoCa, 14 mucinous adenoCa, 15 clear cell adenoCa, 15 endometrioid adenoCa	11 serous cystadenomas, 5 mucinous cystadenomas, 10 serous borderline tumors, 15 muscinous borderline tumors	IHC Western blot	**Ephrin-B1** expression more frequent in high-grade carcinomas High **ephrin-B1** expression correlated with increased reccurence rate and decreased OS	[83]
**ephrin-B2**	72 ovarian cancer samples		IHC Real-time RT-PCR	High **ephrin-B2** expression correlated with clinical stage and low OS No correlation between **ephrin-B2** expr levels and histological type	[82]
**ENDOMETRIUM**					
**EPHA2**	139 endometrial endometrioid carcinoma samples	10 benign endometrial	IHC	High **EPHA2** expression detected in 48% of EECs and 10% of benign samples **EPHA2** overexpression associated with ○ high disease stage ○ high tumor grade ○ increased depth of myometrial invasion ○ low ER and PR expression ○ high ki67 index ○ shorter OS ○ poor outcome	[84]
**EPHB4**	68 endometrial cancer samples	16 normal endometrium samples	IHC Real-time RT-PCR	**EPHB4** expression dominant in cancer cells High **EPHB4** expression levels correlated with ○ increased clinical stage ○ dedifferentiation ○ myometrial invasion ○ low OS	[85]
	11 ER (+)/PR (+) endometrial carcinoma samples, 33 ER (-)/PR (-) endometrial carcinoma samples	10 adenomyosis samples, 2 simple endometrial hyperplasia samples, 20 atypical endometrial hyperplasia samples	IHC Western blot	Overexpression of **EPHB4** detected in er (+)/pr (+) endometrial carcinomas No significant differences in **EPHB4** expression between ER (-)/PR (-) endometrial carcinoma cases and control specimens reported **EPHB4** overexpression associated with er expression	[86]
**ephrin-B2**	68 endometrial cancer samples	16 normal endometrium samples	IHC Real-time RT-PCR	**Ephrin-B2** expression dominant in cancer cells High **ephrin-B2** expression levels correlated with: ○ increased clinical stage ○ dedifferentiation ○ myometrial invasion ○ low OS	[85]
	11 ER (+)/PR (+) endometrial carcinoma samples, 33 ER (-)/PR (-) endometrial carcinoma samples	10 adenomyosis samples, 2 simple endometrial hyperplasia samples, 20 atypical endometrial hyperplasia samples	IHC Western blot	Overexpression of **ephrin-B2** detected in ER (+)/PR (+) endometrial carcinomas No significant differences in **ephrin-B2** expression between ER (-)/PR (-) endometrial carcinoma cases and control specimens reported **Ephrin-B2** overexpression associated with er expression	[86]
**CERVIX**					
**EPHA2**	206 squamous cell cervical cancer samples		IHC	**EPHA2** overexpression correlated with shorter OS	[87]
	217 early squamous cell cervical carcinoma samples		IHC	**EPHA2** expression not associated with OS	[88]
**EPHB2**	20 HGSIL, 53 cervical squamous cell carcinomas	25 normal cervical samples	IHC	**EPHB2** expression correlated with cancer progression, as the percentage of **EPHB2** (+) cells to overall cells was 28% in normal tissues, 40% in HGSIL tissues and 69.8% in SCCs **EPHB2** expression positively correlated with tumor stage	[89]
**EPHB4**	90 cervical carcinoma samples, 15 CIN samples	15 normal cervix samples	IHC	**EPHB4** expression reported higher in cervical cancer and CIN tissues than normal ones **EPHB4** expression correlated with stage and tumor diameter Strong **EPHB4** expression correlated with MVD	[90]
	62 uterine cervical cancer samples		IHC Real-time RT-PCR	**EPHB4** overexpression correlated with: ○ higher stage ○ LN metastasis ○ higher tumor size ○ poor prognosis	[82]
**ephrin-A1**	206 squamous cell cervical cancer samples		IHC	**Ephrin-A1** overexpression correlated with shorter OS	[87]
	217 early squamous cell cervical carcinoma samples		IHC	High **ephrin-A1** staining associated with poor DFS and disease-specific survival	[88]
**ephrin-B2**	90 cervical carcinoma samples, 15 CIN samples	15 normal cervix samples	IHC	**Ephrin-B2** expression reported higher in cervical cancer and CIN tissues than normal ones **Ephrin-B2** expression correlated with tumor diameter Strong **ephrin-B2** expression correlated with MVD	[90]
	62 uterine cervical cancer samples		IHC Real-time RT-PCR	**Ephrin-B2** overexpression correlated with ○ higher stage ○ LN metastasis ○ higher tumor size ○ poor OS	[82]

q real time PCR: quantitative real time transcription-polymerase chain reaction, IHC: immunohistochemistry, TMA: tissue microarrays, OS: overall survival, FIGO: International Federation of Gynaecology and Obstetrics, er: estrogen receptor, pr: progesterone receptor, LN: lymph nodes, Ca: carcinoma, MVD: microvessel density, EOC: epithelial ovarian carcinomas, CIN: cervical intraepithelial neoplasm, HGSIL: high-grade squamous intraepithelial lesion.

### 7.3. Cervix

Two large studies, that each incorporated more than 200 Squamous Cell Carcinoma (SCC) specimens, investigated the protein expression of EPHA2 and its ligand, ephrin-A1, through IHC. The first group reported that EPHA2 overexpression was correlated with shorter patient OS [87], while no such association was reached in the second study [88]. However, both studies supported that high ephrin-A1 expression was correlated with poor patient OS. EPHB2 expression correlated with cancer progression, as the percentage of ratio of EPHB2 positive cells to the number of all cells was 28% in normal tissues, 40% in High Grade Squamous Intraepithelial Lesion tissues and 69.8% in SCCs. Furthermore, EPHB2 was positively correlated with tumor stage [89]. Likewise, EPHB4 and ephrin-B2 expression were reported higher in cervical cancer and Cervical Intraepithelial Neoplasia specimens and associated with tumor diameter, with EPHB4 additionally associated with disease stage. EPHB4 and ephrin-B2 may promote tumorigenesis by driving neoangiogenesis, as their strong protein expression is correlated with MVD [90]. Another study linked EPHB4 and ephrin-B2 overexpression to higher tumor stage, LN metastasis, higher tumor size and poorer prognoses [82]. Table 5 and Figure 5 summarize information on EPH/ephrins and cervical cancer.

## 8. Pediatric Neoplasia

### 8.1. Sarcomas

The limited number of studies on the EPH/ephrin system’s clinical impact on soft tissue tumors concerns Embryonal Rhabdomyosarcoma (ERM) tumors. EPHA2 and ephrin-A1 were reported to be significantly upregulated in ERM tissues compared with normal skeletal muscle specimens [91]. In contrast, EPHB4 overexpression was correlated with positive prognoses (longer OS) for ERM patients [92]. Data are presented on Table 6.

### 8.2. Neuroblastoma

Tang et al [93] investigated, through q PCR, the gene expression of *EPHB6*, *ephrin-B2* and *ephrin-B3* in 50 neuroblastoma tissues, associating it with favorable outcomes (longer OS), in contrast to the negative role of the aforementioned EPH/ephrin members in central nervous system neoplasia [9,12]. The study is summarized in Table 6.

### 8.3. Wilms Tumor

The investigation of EPHB2 gene expression by q PCR in 25 primary Wilms tumors revealed elevated EPHB2 expression in stages 2–4 compared to stage 1 Wilms tumors, probably showing a negative impact on carcinogenesis [94]. The study’s results are presented on Table 6.

**Table 6:** EPHs/ephrins (bold) studied in solid pediatric tumors and correlations with clinicopathological parameters.

EPHs/Ephrins	Malignant Tissues	Benign Control Tissues	Methods	Results	Refs
**RHABDOMYOSARCOMA**					
**EPHA2**	14 ERM samples	normal skeletal muscle samples	real-time RT-PCR	Significant upregulation of **EPHA2** in ERM tissues compared to normal skeletal muscle tissues reported No correlation between **EPHA2** expression and disease state	[91]
**EPHB4**	Not mentioned		Data from the Intergroup Rhabdomyosarcoma Study Group (IRSG)-IV Affymetrix U95 GeneChip database regarding EphB4 expression in human ERMs	**EPHB4** elevated expression correlates with longer OS for ERM patients	[92]
**ephrin-A1**	14 ERM samples	normal skeletal muscle samples	real-time RT-CR	Significant upregulation of **ephrin-A1** in ERM tissues compared to normal skeletal muscle tissues reported No correlation between **ephrin-A1** expression and disease stage	[91]
**NEUROBLASTOMA**					
**EPHB6** **ephrin-B2** **ephrin-B3**	50 neuroblastoma samples		q RT-PCR	High **EPHB6**/**ephrin**-**B2**/**ephrin-B3** expression correlated with longer OS EPHB6 and **ephrin-B3** expression associated with stage **Ephrin-B2** associated with stage and age	[93]
**WILMS TUMOR**					
**EPHB2**	25 primary Wilms tumor samples		q PCR	**EPHB2** expression higher in stage 2–4 Wilms tumors compared with stage 1 tumors	[94]

ERM: embryonal rhabdomyosarcoma, real time RT-PCR: real time reverse transcription-polymerase chain reaction, q RT-PCR: quantitative reverse transcription-polymerase chain reaction.

## 9. Conclusions

EPHs/ephrins seem to heavily impact key molecular steps of carcinogenesis. Their role designates them as possible biomarkers, useful in clinical practice for their accuracy, but also for timely detection of the presence of a neoplasm.

Different members of the EPH/ephrin family carry out distinct actions in different stages of neoplasia. Some members exert their role at early steps of tumorigenesis, aiding tumor cells’ proliferation or enhancing the appropriate cell-matrix interactions that allow cancer cells to locally invade surrounding normal tissues. Others are implicated in the creation of the microenvironment that will allow spreading of the tumor to distant sites, carrying out their action in later disease stages. The distinct roles of the EPH/ephrin system members indicate the diversity of their usefulness in the clinical setting. The expression level evaluation of an individual EPH/ephrin could be utilized to investigate not only the presence of neoplasia, but also the assessment of parameters such as the stage of the disease, the size of the tumor, and the overall disease burden. The EPH/ephrin molecular profile of a tumor should also indicate, in some cases, tumor origin. Moreover, evaluation of the EPH/ephrin molecular signature of a tumor can accurately estimate patients’ prognoses. Determination of fluctuation in their levels could contribute to monitoring treatment efficacy and outcome, as well as to early detection of disease recurrence.

The well-established tumor-promoting or tumor-suppressive properties of the various EPH/ephrin members renders them potential targets of therapeutic interventions. The development of therapeutic agents blocking EPHs/ephrins that enhance carcinogenesis or enhance the expression of those that suppress it represents a promising future treatment strategy. In addition, researchers have proven that different tumors exhibit distinct profiles of EPH/ephrin protein expression, with some cases showing significant upregulation of a certain member and others extensive loss. The colon is a characteristic example, where the majority of EPHs/ephrins seem to have a protective function regarding neoplastic transformation. Most studies underlined that colon cancer tissues exhibit loss of EPHs/ephrins expression of both subgroups A and B [48,49,50,51,52,53,54,55,56,57,58,59,60]. The molecular profile of a tumor could be utilized in order to create a specialized treatment regimen that will benefit each individual patient’s prognosis. Antibodies targeting members of the EPH/ephrin system have already been tested in clinical trials. Anti-EPHA2 monoclonal antibody DS-8895a has been tested on esophageal and gastric cancer patients and EPHA3 antibody IIIA4 (Ifabotuzumab/KB004) on GBM cases and patients with hematologic malignancies. As far as antibody-drug conjugates are concerned, an anti-EPHA2 1C1 antibody was evaluated as an auristatin conjugate, MEDI-547, in patients with solid tumors. Furthermore, anti-ephrin-A4-Calicheamicin (PF-06647263) has been tested on 60 patients, the majority of whom were diagnosed with ovarian or breast cancer [95]. Research has also been conducted towards the creation of a protein compound with the ability to block EPHB4-ephrin-B2 interaction. The final product, sEphB4-HAS, underwent clinical trial that included 70 patients with various malignancies. Four patients exhibited a response to some extent (1 of 3 Kaposi Sarcoma patients, 2 out of 17 head and neck cancer patients and 1 out of 8 hepatocellular cancer patients), half of the patients had stable disease, and the rest demonstrated disease progression [96,97]. The therapeutic agents targeting members of the EPH/ephrin system that have currently undergone clinical testing are summarized on Table 7.

The role of the microenvironment in tumorigenesis on the organ of origin, as well as the spreading of tumor to distant sites, is often overlooked. EPHs/ephrins are among the molecules implicated in interactions between malignant cells and stromal cells that enhance cancer pathogenesis. It is speculated that EPH/ephrin expression on stromal cells promotes invasion of tumor cells in normal surrounding tissues and also provides a positive feedback for malignant cells’ proliferation. EPHA1, EPHA2, EPHA3, EPHA5, EPHB2 and EPHB4 expression has been reported as 2-fold higher in stromal cells isolated from gastric cancer tissues compared with normal gastric tissue stromal cells [44]. Further research on the field of EPH/ephrin stromal cells’ molecular profile is called for, as interrupting those mechanisms of communication between tumor cells and cellular components of surrounding connective tissues could represent possible treatment strategies.

In practice, researchers have established that the varying expression patterns of the EPH/ephrin system heavily impact a multitude of clinicopathological parameters. Large studies that enrolled hundreds of patients revealed the correlation between EPHs/ephrins gene or protein expression and disease stage, histological grade and, most importantly, OS and DFS. In vitro studies reported change in cells’ biological behavior when certain EPH/ephrin genes where either upregulated or silenced. However, many questions remain unanswered regarding the role of the EPH/ephrin family in the molecular pathways of neoplasia. The upregulation of a certain EPH can enhance carcinogenesis in one organ, while suppressing it in a different one [56,57,58,67]. Furthermore, some studies yielded contradictory results regarding the role of EPHs/ephrins in solid tumors. While EPHA1 and EPHA2 showed significantly increased expression in CRC specimens [46,47,48], they were also associated with lower stage tumors [46,47,48], and, in the case of EPHA1, with longer patient OS [48]. Moreover, different groups of researchers reported contradicting results on the effect of a distinct EPH/ephrin in tumorigenesis of an organ, as is the case of EPHA1 in ovarian tumors [75,76]. Such discrepancies underline the need of further research on the subject.

In conclusion, the EPH/ephrin system represents a large family of biomolecules with great possible applications in the fields of diagnosis, prognosis, disease monitoring, and treatment of neoplasia, with an established important clinical impact.

## Figures and Tables

**Table 7:** Agents (bold) targeting EPHs/ephrins tested in clinical trials.

Therapeutic Agent	Target	Category	Malignancy	Reference
**DS-8895a**	EPHA2	Antibody, antagonist	Esophageal and gastric cancer	[95]
**Ifabotuzumab (KB004)**	EPHA3	Antibody, antagonist	Hematologic malignancies	[95]
**MEDI-547**	EPHA2	Antibody-drug conjugate, agonist	Solid tumors (various)	[95]
**PF-06647263**	ephrin-A4	Antibody-drug conjugate, antagonist	Ovarian and breast cancer (majority)	[95]
**sEphB4-HSA**	ephrin-B2	Protein complex, antagonist	Solid and hematologiccancers	[96,97]

## Abbreviations

EPH	erythropoietin-producing human hepatocellular receptors
Ephrin	EPH family receptor interacting proteins
RTK	receptor tyrosine kinases
IHC	immunohistochemistry
TMA	tissue microarrays
PCR	polymerase chain reaction
RT-PCR	reverse transcription-polymerase chain reaction
q PCR	quantitative reverse transcription- polymerase chain reaction
LN	lymph nodes
OS	overall survival
DFS	disease-free survival
MVD	microvessel density
GBM	glioblastoma
ACC	adenoid cystic carcinoma
EMT	epithelial-mesenchymal transition
NSCLC	non-small-cell lung carcinoma
EGFR	epidermal growth factor receptor
CRC	colorectal carcinoma
HCC	hepatocellular carcinoma
PDAC	pancreatic ductal adenocarcinoma
CCRCC	clear cell renal cell carcinoma
TCC	transitional cell carcinoma
PCa	prostate carcinoma
BPH	benign prostate hyperplasia
EOC	epithelial ovarian carcinoma
SCC	squamous cell carcinoma
ERM	embryonal rhabdomyosarcoma
